# Identification and characterization of core abscisic acid (ABA) signaling components and their gene expression profile in response to abiotic stresses in ***Setaria viridis***

**DOI:** 10.1038/s41598-019-40623-5

**Published:** 2019-03-11

**Authors:** Karoline Estefani Duarte, Wagner Rodrigo de Souza, Thaís Ribeiro Santiago, Bruno Leite Sampaio, Ana Paula Ribeiro, Michelle Guitton Cotta, Bárbara Andrade Dias Brito da Cunha, Pierre Roger René Marraccini, Adilson Kenji Kobayashi, Hugo Bruno Correa Molinari

**Affiliations:** 1 Plant Biotechnology Program, Federal University of Lavras (UFLA), Lavras, MG 37200-000 Brazil; 2 Genetics and Biotechnology Laboratory, Embrapa Agroenergy (CNPAE), Brasilia, DF 70770-901 Brazil; 3 Centro de Ciências Naturais e Humanas, Universidade Federal do ABC (UFABC), São Bernardo do Campo, Santo André, SP 09606-045 Brazil; 4 Department of Cell Biology, University of Brasília (UnB), Brasília, DF 70910-900 Brazil; 5 CIRAD, UMR AGAP (University Montpellier, CIRAD, IRD, INRA), Montpellier, 34398 France; 6 CIRAD, UMR IPME (University Montpellier, CIRAD, IRD, Montpellier), Agricultural Genetics Institute, LMI RICE2 Hanoi, Vietnam

## Abstract

Abscisic acid (ABA) is an essential phytohormone that regulates growth, development and adaptation of plants to environmental stresses. In Arabidopsis and other higher plants, ABA signal transduction involves three core components namely PYR/PYL/RCAR ABA receptors (PYLs), type 2C protein phosphatases (PP2Cs) and class III SNF-1-related protein kinase 2 (SnRK2s). In the present study, we reported the identification and characterization of the core ABA signaling components in *Setaria viridis*, an emerging model plant for cereals and feedstock crops presenting C4 metabolism, leading to the identification of eight *PYL* (*SvPYL1* to 8), twelve *PP2C* (*SvPP2C1* to 12) and eleven *SnRK2* (*SvSnRK2*.1 through *SvSnRK*2.*11*) genes. In order to study the expression profiles of these genes, two different *S*. *viridis* accessions (A10.1 and Ast-1) were submitted to drought, salinity and cold stresses, in addition to application of exogenous ABA. Differential gene expression profiles were observed in each treatment and plant genotype, demonstrating variations of ABA stress responses within the same species. These differential responses to stresses were also assessed by physiological measurements such as photosynthesis, stomatal conductance and transpiration rate. This study allows a detailed analysis of gene expression of the core ABA signaling components in *Setaria viridis* submitted to different treatments and provides suitable targets for genetic engineering of C4 plants aiming tolerance to abiotic stresses.

## Introduction

Abscisic acid (ABA) is a phytohormone involved in the control of many aspects of plant growth and development including embryo maturation, cell division and elongation, seed dormancy and germination, root growth and floral induction^1–5^. In addition, ABA also responds to a variety of environmental stresses, including biotic and abiotic stresses such as drought, cold and salinity^6–8^. Chemically, ABA is a sesquiterpene synthesized in plants from β-carotene, via multiple enzymatic reactions that involves zeaxanthin oxidase (ZEP), 9-*cis*-epoxycarotenoid dioxygenase (NCED), ABA-aldehyde oxidase (AAO) and molybdenium cofactor sulfurase^9^ (MCSU). In abiotic stress conditions, ABA levels increase, leading to a signaling cascade that ultimately activates plant adaptation responses to stress^10^. The ABA-activated signaling network was recently unraveled, and the identification of ABA receptors is among the most important advances in stress signaling in the past decade^4,11^.

ABA receptors were first uncovered in *Arabidopsis*, where three core components have been identified: the ABA receptor PYR/PYL/RCAR (PYL) protein family, the negative regulator type 2C protein phosphatase (PP2C) and the positive regulator class III SNF-1-related protein kinase 2 (SnRK2). Some of these receptors were also identified in other plant species such as sorghum, maize and rubber tree^12–14^. PYR proteins were identified through genetic analyses, which found that PYR1 (*Pyrabactin Resistance 1*) and members of its 13 relative proteins (*Pyrabactin Resistance 1-Like*; PYL) are necessary for proper ABA signal transduction in *Arabidopsis*^15,16^. This study also demonstrated that PYR1 binds to ABA and inhibits the group A protein phosphatases 2Cs (clade A PP2Cs), whose members include ABI1, ABI2 (ABA-insensitive 1 and 2) and HAB1 (*Hypersensitive to ABA 1*). The genetic evidences suggest that PP2Cs act as negative regulators of ABA-dependent pathways, and this function appears to be conserved from *Arabidopsis* to moss^17^. The main targets of PP2Cs identified to date are related to protein kinases involved as positive regulators of ABA signaling^4,17^. Among these kinases, the class III SNF-1-related protein kinases 2 (SnRK2s) are the most implicated in positive regulation of ABA signaling, especially because of the strong phenotype observed in the *Arabidopsis* triple mutant *snrk2*.*2/2*.3*/2*.*6*, which could germinate and grow on 50 µM ABA, an abnormal phenotype demonstrated by ABA-insensitive mutants^18^. Some evidences showed that SnRK2s might directly phosphorylate members of the ABF/AREB/ABI5 clade of bZIP transcription factors, promoting ABA-induced gene expression^18–21^. In summary, ABA binds to a PYL protein, resulting in inhibition of PP2Cs through the ABA-PYL-PP2C complex. This complex leads to accumulation of phosphorylated SnRK2s, which leads to phosphorylation of ABA-responsive element binding factors (ABFs) and subsequent ABA gene expression for appropriate cellular responses^4^.

Some important crops used as sources of food and feedstock belong to the Panicoideae subfamily and include cereal grains and grasses such as sugarcane (*Saccharum* spp.), maize (*Zea mays*), sorghum (*Sorghum bicolor*) and switchgrass^22^ (*Panicum virgatum*). Abiotic stresses such as cold, drought and salinity are among the most deleterious environmental stresses in these crops, responsible for great yield losses worldwide^23^. Despite the advancements achieved in these crops towards the comprehension of the molecular and biochemical pathways associated with abiotic stresses, the complexity of the genome and the long generation times required have hindered the progress of these studies. In this context, *S*. *viridis* has emerged as a suitable C4 model species for molecular and genetic studies. It is a short, fast-growing, C4 metabolism plant, with its genome sequence available, making it a suitable model plant for genetic studies^24,25^. Moreover, *S*. *viridis* is highly responsive to *Agrobacterium tumefaciens*-mediated genetic transformation, with well-established *in vitro* transformation protocols^24,26^ and more recently, spike-dipping transformation methods have also been proposed^27,28^. Genetically engineered *S*. *viridis* plants can be used in a proof-of-concept approach to evaluate phenotypes related to important agricultural traits such as abiotic stress tolerance, resistance to pathogens and improved yield and biomass^29–31^. The tested promising genes could be further transferred to a target crop.

In the present study, the identification and characterization of the ABA receptors *PYL* and the core signaling components *PP2C* (clade A) and *SnRK2* gene families in *S*. *viridis* is reported. Since phenotypic variability in natural accessions of *S*. *viridis* has been reported^28,32,33^, two different *S*. *viridis* accessions (A10.1 and Ast-1) was used to study gene expression of core ABA signaling components under drought, salinity, cold and exogenous ABA application. A total of 8 PYLs (*SvPYL1* to *SvPYL* 8), 12 PP2Cs (*SvPP2C1* to *SvPP2C 12*) and 11 SnRK2s (*SvSnRK2*.*1* to *SvSnRK2*.*11*) were found in *S*. *viridis* genome. Differential gene expression was found for the different treatments and accessions, demonstrating that even within the same species the abiotic stress responses can be variable. Gas exchange measurements also demonstrated that the two accessions studied have slightly different responses to abiotic stresses. This study provides suitable targets for genetic engineering of C4 plants aiming tolerance to abiotic stresses.

## Results

### Genome-wide identification and characterization of the core ABA signaling components in *S*. *viridis*

The search for *PYR/PYL*, *PP2C* and *SnRK2* genes in *S*. *viridis* genome was performed using two different strategies. For both strategies applied, similar number of genes was found. Based on amino acid sequences of *S*. *viridis*, eight, twelve and eleven putative genes of *PYR/PYL*, *PP2C* and *SnRK2* were identified, respectively (Supplementary Fig. S1), and described separately below. The complete set of gene orthology for *Setaria viridis* (Sv) *PYLs*, *PP2Cs* and *SnRK2s*, in comparison with *Arabidopsis thaliana* (At), *Oryza sativa* (Os) and *Sorghum bicolor* (Sb) is presented in Supplementary Table S1.

#### SvPYLs

**Figure 1:**
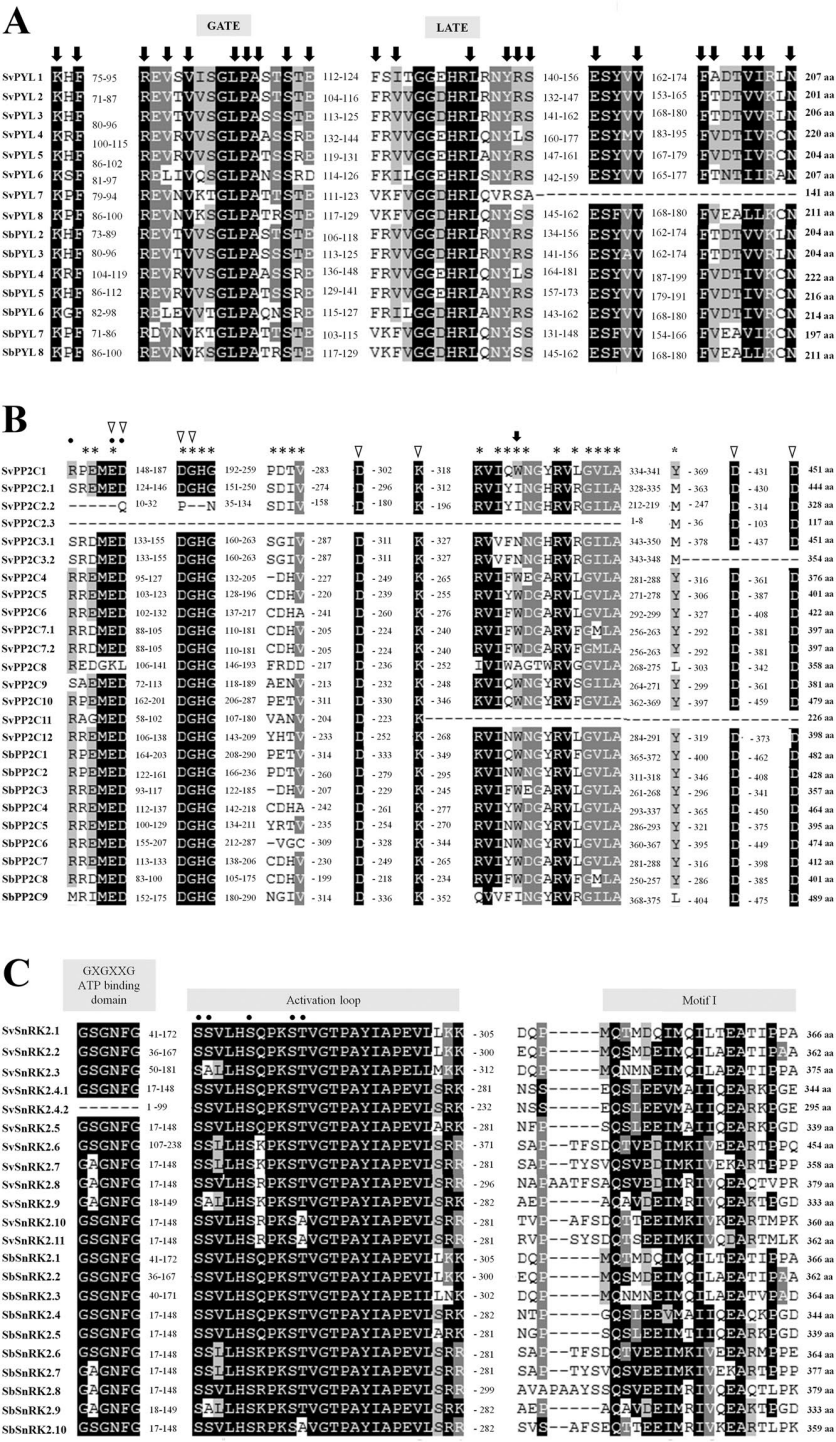
Sequence alignment of *S*. *viridis* SvPYL, SvPP2C and SvSnRK2 proteins and SbPYL, SbPP2C and SbSnRK2 from *Sorghum bicolor*. For each protein, total length is indicated in amino acids (aa). Conserved residues are shaded with black or grey. (**A**) Sequence alignment of eight characterized SvPYLs and x SbPYLs receptors. Conserved residues and residues involved in ligand binding in ABA receptors are marked by black arrows, and the Gate and Latch domains are indicated. (**B**) Sequence alignment of the 12 SvPP2Cs and the nine characterized SbPP2Cs. Residues involved in interaction with ABA, PYLs and Mg^2+^/Mg^2+^ ions are marked by black arrows, asterisks and triangles, respectively. Phosphatase sites are marked with black points. (**C**) The subclass III SvSnRK2 amino acid sequences are compared with the predicted amino acid sequences of SbSnRK2s. The functional domains ATP binding site, activation loop and motif I are indicated. The phosphatase sites are marked with black points.

The identified *PYR/PYL* genes were designated as *SvPYL1* through *SvPYL8*. The size of their corresponding SvPYL proteins ranged from 141 to 220 amino acids (aa), with molecular weight (MW) from 15.11 to 23.67 KDa and pI from 5.24 to 8.88 (Table 1). They contained the polyketide cyclase 2 domain (PF10604) localized between the positions 43–213 aa (Fig. 1A). It was also possible to identify in SvPYL proteins a conserved motif 1 related to the well-known “GATE” and “LATCH” loop regions and motifs 2–3 involved in ABA binding (Fig. 1A).

**Table 1:** List of *SvPYL*, *SvPP2C* and *SvSnRK2* genes identified in *Setaria viridis*.

Gene Name	Chr	Start	Final	aa	pI	MW	PFAM	GeneBank ID
*SvPYL1*	9	34761413	34763538	207	5.24	22.13	48–192	MG766913
*SvPYL2*	*1*	*2710051*	*2713429*	*201*	*5*.*97*	*21*.*75*	*43–180*	*MG766914*
*SvPYL3*	4	35821353	35821973	206	6.71	22.13	52–198	MG766907
*SvPYL4*	9	46321820	46323300	220	6.62	22.93	70–213	MG766908
*SvPYL5*	3	15949957	15951675	204	8.88	21.58	56–197	MG766909
*SvPYL6*	5	39467969	39468592	207	6.75	22.70	51–194	MG766910
*SvPYL7*	3	5043188	5045597	141	8.74	15.11	47–131	MG766912
*SvPYL8*	1	1156602	1160325	211	5.92	23.67	55–198	MG766911
*SvPP2C1*	3	8481733	8485238	451	4.99	47.10	176–434	MG766927
*SvPP2C2*.*1*	6	387891	391296	444	5.03	47.08	113–396	MG766928
*SvPP2C2*.*2*	6	387891	391296	328	5.24	35.25	132–281	MG766929
*SvPP2C2*.*3*	6	389629	391657	117	4.35	12.47	1–104	MG766930
*SvPP2C3*.*1*	7	785451	790598	451	5.30	47.17	119–410	MG766931
*SvPP2C3*.*2*	7	786886	790598	354	6.72	36.81	116–354	MG766932
*SvPP2C4*	2	25703196	25705262	376	6.80	39.38	78–364	MG766933
*SvPP2C5*	5	40142449	40144716	401	5.87	42.51	87–390	MG766934
*SvPP2C6*	3	16922545	16924778	422	7.05	43.93	85–411	MG766935
*SvPP2C7*.*1*	9	47906442	47908435	397	5.69	42.35	71–330	MG766936
*SvPP2C7*.*2*	9	47906340	47908359	397	5.69	42.35	71–330	MG766937
*SvPP2C8*	1	1190419	1194718	358	5.79	38.52	105–345	MG766938
*SvPP2C9*	3	11944691	11947945	381	5.28	40.63	58–364	MG766939
*SvPP2C10*	5	25838987	25843846	479	4.73	49.71	176–462	MG766940
*SvPP2C11*	5	33933094	33934782	226	6.08	23.68	44–226	MG766941
*SvPP2C12*	3	9748111	9750887	398	8.50	41.23	89–376	MG766942
*SvSnRK2*.*1*	9	4714220	4719224	366	4.81	41.48	28–284	MG766915
*SvSnRK2*.*2*	9	11263105	11266229	362	4.73	40.73	23–279	MG766916
*SvSnRK2*.*3*	3	46484835	46487553	375	4.94	41.55	37–293	MG766917
*SvSnRK2*.*4*.*1*	9	41982274	41986678	344	5.10	39.13	4–260	MG766918
*SvSnRK2*.*4*.*2*	9	41982637	41986678	295	4.94	33.43	2–211	MG766919
*SvSnRK2*.*5*	2	44725651	44730688	339	5.30	38.47	4–260	MG766920
*SvSnRK2*.*6*	1	26704997	26709453	454	8.30	51.67	94–350	MG766921
*SvSnRK2*.*7*	7	19178334	19183417	358	6.00	40.99	4–260	MG766922
*SvSnRK2*.*8*	3	274209	277259	379	5.99	43.05	4–260	MG766923
*SvSnRK2*.*9*	9	35394567	35397834	333	5.40	37.90	5–261	MG766924
*SvSnRK2*.*10*	3	18332249	18337532	360	5.68	41.77	4–260	MG766925
*SvSnRK2*.*11*	5	41261960	41266866	362	6.06	42.34	4–260	MG766926

Chr: Chromosome location; aa: length of amino acids; pI: isoelectric point; MW: molecular weight.

**Figure 2:**
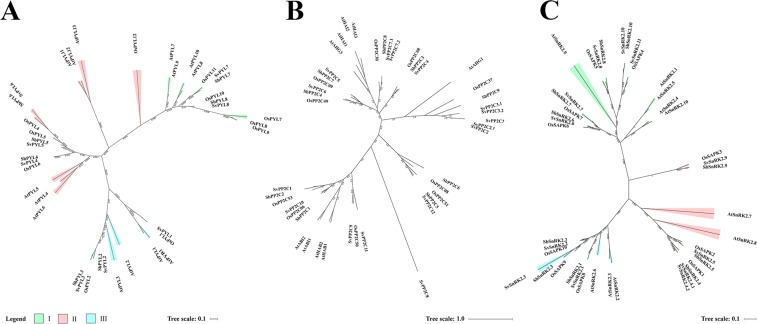
Phylogenetic analysis of ABA core signaling protein components from *Setaria viridis*. Maximum likelihood phylogeny of functionally characterized (**A**) SvPYLs (**B**) SvPP2Cs (**C**) SvSnRK2s proteins and their close homologs from *Arabdopsis thaliana (At)*, *Sorghum bicolor* (*Sb*) and *Oryza sativa* (*Os*) are shown. The phylogenetic tree was constructed using FastTree 2.1.5 program. Branch color scale represents SH-like local support (red to lower values and green to higher values).

**Figure 3:**
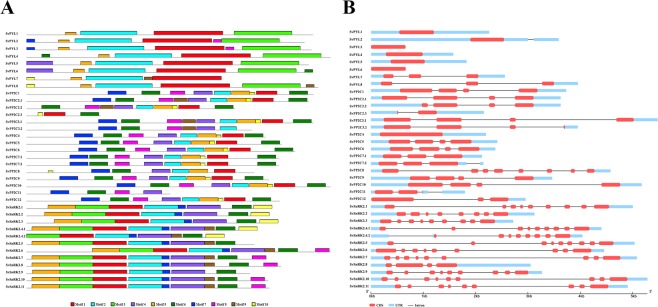
(**A**) MEME analysis of 10 conserved motifs of SvPYLs, SvPP2Cs and SvSnRK2s proteins. Different color boxes correspond to different motifs. (**B**) Gene structure of ABA core signaling components. Filled boxes and single lines show exons and introns, respectively.

A phylogenetic analysis was performed based on similarities to PYL proteins from *Arabidopsis* (AtPYLs), *Oryza sativa* (OsPYLs) and *Sorghum bicolor* (SbPYLs), which divided SvPYLs in subfamilies I, II and III (Fig. 2A). SvPYL1–3, SvPYL4-6 and SvPYL7-8 were classified as subclass III, II and I, respectively. With the exception of SvPYL7, all identified SvPYL proteins have orthologs in *Arabidopsis* (Supplementary Table S1). Intron-exon analysis of *SvPYL* genes showed that only genes clustered into subfamily I have 2 introns while those from subfamilies II and III have none intronic regions (Fig. 3B).

#### SvPP2Cs

In total, 12 *PP2C* genes, designated as *SvPP2C1* to *SvPP2C12*, were identified in *S*. *viridis* genome (Fig. 2B). Three isoforms were observed for *SvPP2C2* (*SvPP2C2*.*1*, *SvPP2C2*.*2* and *SvPP2C2*.3) while two isoforms were found for *SvPP2C3* (*SvPP2C3*.*1* and *SvPP2C3*.*2*) and *SvPP2C7* (*SvPP2C7*.*1* and *SvPP2C7*.*2*). The size of SvPP2C proteins ranged from 117 (SvPP2C2.3) to 479 (SvPP2C10) amino acids with MW between 12.47 to 49.71 KDa and pI ranging from 4.35 to 8.50 (Table 1). Based on Pfam analysis, a protein domain PF00481 was identify and conserved in all putative SvPP2C proteins. Furthermore, binding residues for PYL and the cofactors Mn^2+^/Mg^2+^ in the motifs 2, 3, 5 and 7 were also identified in SvPP2Cs (Fig. 3A). With the exception of SvPP2C2.2, SvPP2C2.3, SvPP2C3.2 and SvPP2C11, SvPP2Cs sequences contain well-characterized functional residues and domain regulators of ABA necessary to interaction with PYLs and SnRK2s proteins (Fig. 1B). Analysis of proteins demonstrated that SvPP2Cs have orthologs mainly in *O*. *sativa* (OsPP2Cs) and *S*. *bicolor* (SbPP2Cs), with exception to SvPP2C8 and SvPP2C11 (Supplementary Table S1). Intro-exon analysis showed a variable numbers of introns in *SvPP2C* genes, with the predominance of three introns in the majority of the genes (Fig. 3B).

#### SvSnRK2s

Based on the presence of Pfam domain PF0069 and similarity with query sequences, 11 non-redundant *SnRK2* genes (named from *SvSnRK2*.*1* to *SvSnRK2*.*11*) were found in *S*. *viridis* genome. *SnRK2*.*4* was the only gene presenting two isoforms, (named *SvSnRK2*.*4*.*1 and SvSnRK2*.*4*.*2*). The length of putative SvSnRK2 proteins ranged from 295 to 454 aa, with MW from 33.43 to 51.67 KDa and pI from 4.73 to 8.30 (Table 1). Except for SvSnRK2.4.2 protein, which lacks the ATP-binding loop domain, all these phosphatases contained the five important conserved motifs, including: (1) the ATP-binding domain (motif 5), (2) the activation loop (motif 2), (3) the PP2C interface residues (also called SnRK2 box), (4) the motif I (motif 6) and (5) ABA box domains, (Figs 1C and 3A). The bootstrap values deduced from the phylogenetic analysis revealed that SvSnRK2s were divided into subclasses I, II and III (Fig. 2C). The Subclass I includes *SvSnRK2*.*6*, *SvSnRK2*.*7*, *SvSnRK2*.*8*, *SvSnRK2*.*10* and *SvSnRK2*.*11*, with the predominance of eight introns in their corresponding genes (Fig. 3B). The *SvSnRK2*.*4*, *SvSnRK2*.*5* and *SvSnRK2*.*9*, containing mostly eight introns, were classified into subfamily II (Fig. 3B). Finally, subfamily III comprised the genes *SvSnRK2*.*1*, *SvSnRK2*.*2* and *SvSnRK2*.*3*, with genes containing 7 to 8 introns (Fig. 3B). Orthologs of *SvSnRK2s* genes were found in *S*. *bicolor* and/or *O*. *sativa* (Supplementary Table S1 and Fig. 2C).

**Figure 4:**
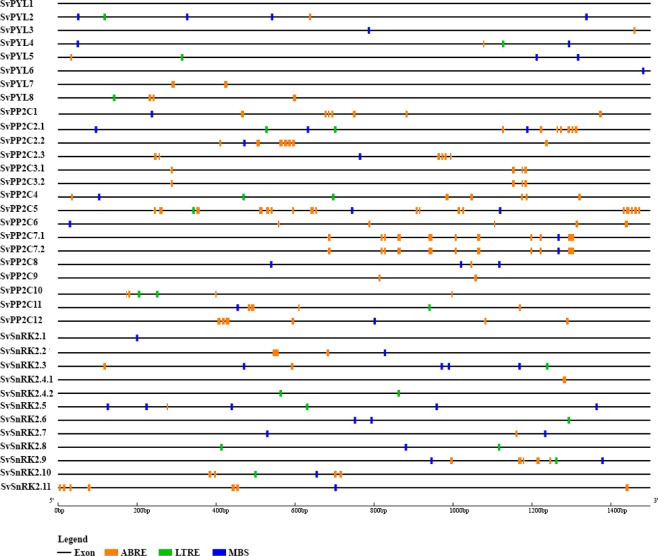
*Cis*-elements in the putative promoter regions of *SvPYL*, *SvPP2C* and *SvSnRK2* genes related with ABA-responsive element (ABRE), low temperature-responsive element (LTRE) and MYB binding site (MBS).

Analysis of *cis*-acting regulatory elements (CARE) of putative promoter regions in *SvPYL*, *SvPP2C* and *SvSnRK2* genes was performed using PlantCARE database. With exception to *SvPYL1* gene, the putative promoter regions for remaining (n = 7) *SvPYL* genes analyzed presented at least one of the following DNA binding domains: MYB binding site (MBS), low temperature responsive element (LTRE) and ABA-responsive element (ABRE) (Fig. 4). The ABRE DNA motif was predominant in the promoter region of *SvPYL* subfamily I (*SvPYL7-8*), with at least four of these *cis*-elements present in these sequences. Regarding the subfamily II, MYB, LTRE and ABRE were found in *SvPYL4* and *SvPYL5* promoter regions while one MBS was found in *SvPYL6* promoter region.

The majority of the putative promoter regions of *SvPYLs*, *SvPP2Cs* and *SvSnRK2s* genes analyzed contains the ABRE element. This DNA motif was present up to 18 times in the promoter region of *SvPP2C5* gene (Fig. 4). Regarding the *SvSnRK2* promoter regions, at least two different CAREs were found, except in *SvSnRK2*.*1*, *SvSnRK2*.*4*.*1* and *SvSnRK2*.*4*.*2* promoter regions. The MBS element was not detected only in *SvSnRK2*.*4* promoter region (Fig. 4).

### Physiological responses of two natural accessions of *S*. *viridis* to abiotic stresses and exogenous ABA application

Physiological plasticity was already reported for different accessions of *S*. *viridis* that occur naturally in different locations of the world^28,32,33^. The physiological responses of two *S*. *viridis* accessions (A10.1 and Ast-1) under well-watered conditions and submitted to drought, salinity, cold and exogenous ABA application were evaluated. The accession A10.1 is originated from United States and it is frequently used for genetic transformations^24,26–28^, while the accession Ast-1 originates from Azerbaijan (GRIN, USDA, www.ars-grin.gov) (Supplementary Fig. S2).

**Figure 5:**
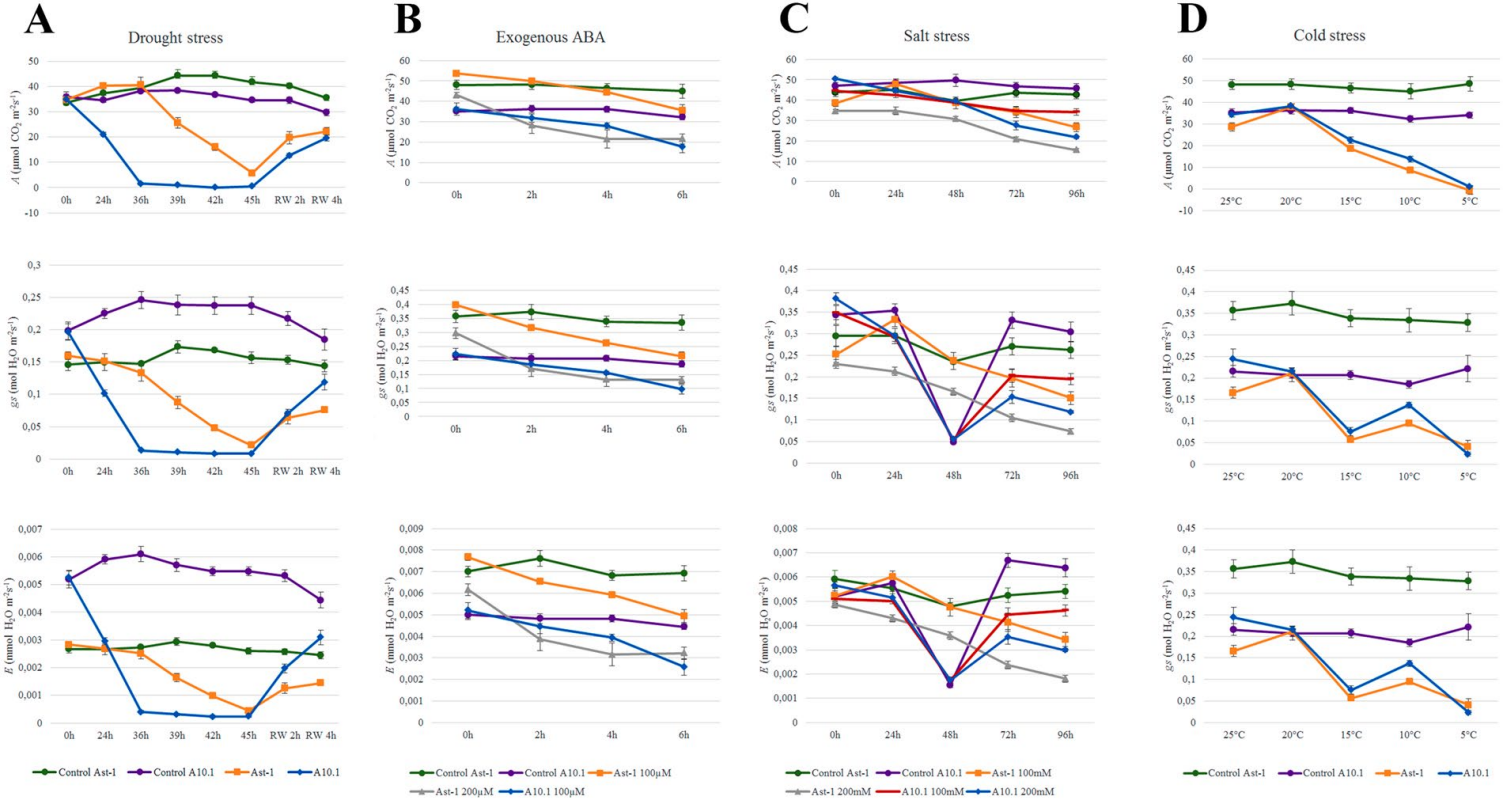
Physiological responses of *Setaria viridis* accessions submitted to abiotic stresses and exogenous ABA treatment. Photosynthesis (**A**), stomatal conductance (*g*_*s*_) and transpiration rate (**E**) are shown for drought (**A**), exogenous ABA application (**B**), salt stress (**C**) and cold stress (**D**). RW refers to plant rewatering after the drought stress period. The statistical analysis was performed and presented in the Supplementary Tables S2–S5.

Gas exchange measurements revealed that physiological responses of A10.1 and Ast-1 to drought stress and exogenous ABA application were slightly different (Fig. 5A,B). In drought conditions, A10.1 achieved the minimum photosynthetic rate (*A*) after 36 h of water deprivation, while the Ast-1 reached the minimum *A* after 45 h (Fig. 5A). In addition, Ast-1 plants were able to re-establish ~65% of the initial photosynthesis 4 h after rehydration, while A10.1 plants could re-establish ~55% of initial *A* in the same condition (Fig. 5A). These results suggest that, in our experimental conditions, Ast-1 was slightly more resistant to dehydration than A10.1. Interestingly, the photosynthetic rate of Ast-1 plants was not affected after 24 h of drought, but the stomatal conductance (*g*_*s*_) and the transpiration rate (*E*) decreased at this time point (Fig. 5A). Exogenous application of ABA had different effects on A10.1 and Ast-1 plants. As 100 µM ABA was able to inhibit *A* by ~40% in A10.1 plants, only ABA concentration levels at 200 µM were able to cause a significant *A* inhibition in Ast-1 plants, suggesting that the accession A10.1 could be more responsive (sensitive) to ABA when compared to Ast-1 (Fig. 5B). Under salinity and cold conditions, *A*, *g*_*s*_ and *E* were similar between the accessions (Fig. 5C,D). In both accessions, 100 mM NaCl treatment caused an inhibition of ~30% of *A* after 96 h, while 200 mM NaCl inhibited *A* by ~60% of (Fig. 5C). In our experiments, A10.1 and Ast-1 plants were grown in controlled conditions at 25 °C. The gradual decrease of temperature induced a linear inhibition of *A*, with the minimum *A* rate achieved at 5 °C in both accessions. However, during cold treatment *g*_*s*_ and *E* presented a bell-shaped curve response, decreasing at 15 °C and 5 °C, but increasing at 10 °C in both accessions (Fig. 5D).

### Gene expression profile of putative core ABA signaling components in *S*. *viridis* accessions submitted to different abiotic stresses

To investigate the expression pattern of *S*. *viridis PYL*, *PP2C and SnRK2* genes, qRT-PCR experiments were performed in leaves of A10.1 and Ast-1 plants submitted to drought, salinity, cold and exogenous ABA application. The tissues were collected during the time course of the treatments, based on the physiological responses observed previously (Fig. 5). All data represent the fold-change in expression of genes when compared to non-stress conditions at the beginning of the experiment (0 h). In this study, low, moderate and strong up- or downregulation were designated as 2–3, 4–10 and >10-fold change of expression related to controls, respectively (Supplementary Fig. S3).

### Drought stress

**Figure 6:**
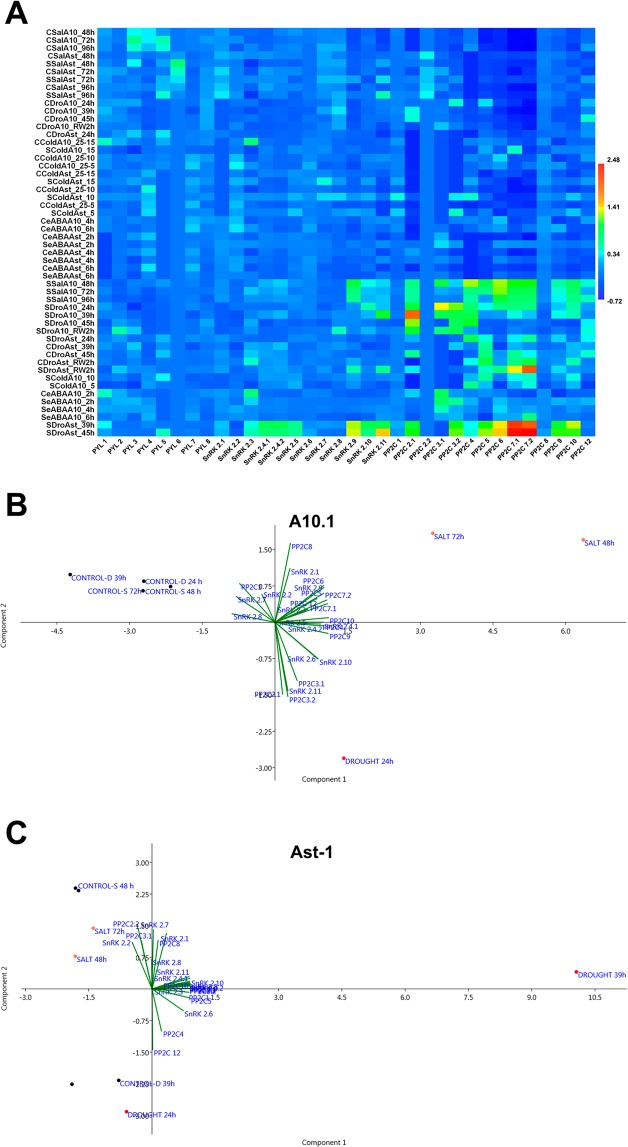
Multivariate analysis of gene expression profile in different abiotic stresses in A10.1 and Ast-1 accessions of *Setaria viridis*. (**A**) Heat map of expression profile of *SvPYL*, *SvPP2C* and *SvSnRK2* genes for all treatments (drought, salt, cold and exogenous ABA). (**B**) Principal component analysis (PCA) exhibiting the correlation between treatments (drought and salt) and *SvPP2C* and *SvSnRK2* for the accession A10.1. (**C**) Principal component analysis (PCA) exhibiting the correlation between treatments (drought and salt) and *SvPP2C* and *SvSnRK2* for the accession Ast-1. Control-C, Stressed-S, Drought-Dro, salt-Sal, cold-Col and exogenous ABA-eABA.

For drought treatment, the transcripts were analyzed at 0 (control), 24, 39 and 45 h after stress application, with differential expression patterns observed between the two accessions (Fig. 6A). In the results obtained for A10.1 low levels of expression of the *SvPYL4* and 7 were observed, while the expression of *SvPYL1*, *2*, *3* and *8* was significant in control conditions. Overall, drought stress downregulated the expression of *SvPYLs*, whereas the expression and *SvPYL2* and *3* greatly increased after re-watering, when the transcript levels were compared to the last time point of drought (Supplementary Fig. S4). For Ast-1 downregulation of *SvPYL3* and upregulation of *SvPYL1*, *4*, and 7 was observed. Expression of *SvPYL1* increased after 39 h of drought stress, however, the levels decreased when the photosynthesis rate reached its minimum (Fig. 5A). During re-watering of Ast-1 plants, expression of *SvPYL2* and 4 increased slightly when compared to dehydrated plants (Figs 6A and S4).

Concerning the ABA core signaling components, all *SvSnRK2s* (except *SvSnRK2*.*1*) and all *SvPP2Cs* (except *SvPP2C2*.*2*, *11* and 12), responded to drought in at least one time point in A10.1 plants (Figs 6A and S4). In this accession, *SnRK2* genes showed differential expression patterns, some of them upregulated (like *SvSnRK2*.*3*, *4*.*1/4*.*2*, 5, 6, 9, 10 and 11) and the others slightly down-regulated (i.e. *SvSnRK2*.*2*, 7 and 8), mainly in early (24 h) or late (45 h) stages of drought. Regarding the *PP2C* genes of group A, expression of *SvPP2C1* and *SvPP2C8* decreased slightly in A10.1 plants in early stages of drought treatment, while *SvPP2C2*.*1*, *3*.*1/3*.*2*, 4, 5, 6, *7*.*1/7*.*2*, 9 and 10 genes were upregulated. In Ast-1, with exception to *SvSnRK2*.*7* and 8, and excepting *SvPP2C3*.*1/3*.*2*, *8* and 12 genes, the expression profile of *SvSnRK2* and *SvPP2C* genes were significantly upregulated in response to drought in at least one time point (Supplementary Fig. S4). In addition, the expression of most *SvSnRK2* and *SvPP2C* genes was upregulated under drought conditions and downregulated during re-watering.

### Salt treatment

For salt treatment, the transcripts were analyzed 48, 72 and 96 hours after the addition of 200 mM NaCl, which was the salt concentration that significantly affected the physiological traits in both accessions (Fig. 5C).

Except *SvPYL* genes, A10.1 plants largely activated the core ABA signaling genes during salt stress (Supplementary Fig. S3). For this accession, the expression of *SvSnRK2*.*3*, *4*.*1/4*.2, 9, 10, 11 genes and of all *SvPP2C* genes was upregulated in 48 h of salt treatment, excepted for *SvPP2C1* and *2*.*2* genes. In addition, expression of *SvSnRK2*.*7* and 8 was downregulated under salt stress. On the other hand, expression of *SvPYL3*, 5 and 6, *SnRK2*.*8*, *9* and *11*, and *SvPP2C2*.*1*, *3*.*2* and 12 genes was slightly upregulated in at least one time point under salt treatment in Ast-1 plants (Supplementary Fig. S5).

### Cold Treatment

For cold treatment transcript analysis, the tissues were collected 24 h after each selected temperature was reached (15, 10 and 5 °C). In A10.1 plants under cold stress, *SvPYL1*, 2, 3, 7 and 8 were downregulated (Supplementary Fig. S6). At temperatures below 15 °C, transcripts of *SvSnRK2*.*4*.*1/4*.*2*, *5* and 9 genes were slightly upregulated while *SvPP2C4*, *5*, *7*.*1/7*.*2* and 10 genes were strongly upregulated. In Ast-1 plants, *SvPYL* genes showed low response to cold, with weak upregulation of *SvPP2C3*.*2* and 8 (Supplementary Fig. S3). Overall, *SvSnRK2* genes were upregulated (~2–3 fold) during cold stress (Supplementary Fig. S3). Interestingly, the isoforms *SvSnRK2s* 4.*1* and *4*.*2* were downregulated in Ast-1 plants at 15 °C but the decrease in temperature led to the upregulation of these genes (Supplementary Fig. S6). Moreover, 8 out of 14 *SvPP2C* genes in Ast-1 plants under cold stress were slightly upregulated, but *SvPP2C7*.*1* showed the same pattern as *SvSnRK4*.*1/4*.*2*, being downregulated at 15 °C and upregulated at lower temperatures (Supplementary Fig. S6).

### Treatment by exogenous ABA

Expression levels of genes of ABA signaling components were analyzed in plants under exogenous ABA treatment. The tissues were collected after 2, 4 and 6 h after ABA application at 100 µM and 200 µM, according to physiological responses observed for A10.1 and Ast-1 plants, respectively (Fig. 5B). *PYL* receptor genes responded differently between A10.1 and Ast-1, as observed in Supplementary Fig. S3.

In A10.1 accession, *SvPYL8* was significantly downregulated after ABA application, while in Ast-1 significant downregulation was observed for *SvPYL3* and *4*. In A10.1 plants, *SvPYL4* was upregulated in a time course-dependent manner under ABA treatment, while *SnRK2*.*3* was strongly downregulated after 2 h of exogenously applied ABA (Supplementary Fig. S7). However, *SvSnRK2*.*3* was weakly upregulated in Ast-1 accession in the same time, demonstrating contrasting effects of ABA application among the accessions. The *PP2C* genes were mostly upregulated in both accessions, especially after 2 h of ABA application (Supplementary Fig. S7).

### Multivariate analysis of the gene expression profile of core ABA signalling components under abiotic stresses

We performed hierarchical cluster analysis (HCA) and principal component analysis (PCA) in order to obtain a more comprehensive understanding of the expression profile of genes comprising the core ABA signalling components under different treatments (Supplementary Fig. S8). After the loading of the whole gene expression data comprising all families studied, it was observed negative coefficient values for *SvPYL* gene family (Supplementary Fig. 8C), indicating low level of influence of this family under our experimental conditions. When the *SvPYL* family was included in the HCA, it was verified two major groups (Supplementary Fig. 8A), one mostly comprising control samples (represented by the red group in Supplementary Fig. 8A) and another group comprising samples submitted to treatments (represented by the yellow group in Supplementary Fig. 8A). The PCA analysis revealed that *PP2Cs* genes are the most responsive components during salt and drought stresses (Supplementary Fig. 8B). In the analysis containing *SvPYL* gene family, however, we could not observe clear differences in the gene expression profile between the accessions A10.1 and Ast-1 and the different treatments applied. Thus, we decided to perform HCA and PCA studies excluding the data referred to *SvPYL* gene family. Using this approach, we were able to observe three major groups in the HCA (Supplementary Fig. 8D), where one of the groups mostly represents samples of Ast-1 accession, while the other two major groups represented the studies in A10.1 accession. The PCA analysis performed after exclusion of the *SvPYL* gene family data also showed that *PP2C* genes were very responsive to drought and salt stresses (Supplementary Fig. 8E). A heat-map was constructed to demonstrate the influence of the different treatments in the expression of *SvPYL*, *SvSnRK2* and *SvPP2C* genes in both accessions (Fig. 6A). The heat-map clearly showed that only few members of *SnRKs* and *PP2Cs* were the most expressed genes in response to salt and drought conditions, especially in A10.1 accession. Moreover, our data also demonstrated that gene expression of the core ABA signalling components decreases in a time-dependent manner, suggesting that rapid ABA responses are achieved in *S*. *viridis* under abiotic stresses.

These results prompted us to perform a multivariate analysis correlating only the data corresponding to the expression of *SnRK*s, *PP2Cs* and the osmotic stresses, to rationalize which genes were expressed in each condition and if there were any significant differences between the accessions. As demonstrated in Fig. 6B,C, we observed clear differences in gene expression between A10.1 and Ast-1 genotypes submitted to salt and drought stresses. The genotype A10.1 was more responsive to the stress, and three major groups could be identified in the PCA, corresponding to controls, salt and drought (Fig. 6B). The most expressed genes in A10.1 submitted to salt stress were *SnRKs 2*.*1*, *2*.*9*, *2*.*3* and *PP2Cs 8*, 6, 5, *7*.*1* and *7*.*2*, while *SnRK*s *2*.*6*, *2*.*10*, *2*.*11* and *PP2C*s *3*.*1* and *3*.*2* were the most expressed genes in A10.1 submitted to drought. The accession Ast-1 appeared to be less responsive to osmotic stresses, as we could not observe a clear separation in the groups correlating gene expression versus controls and treatments (Fig. 6C).

### ABA accumulation in *S*. *viridis* submitted to abiotic stresses

**Figure 7:**
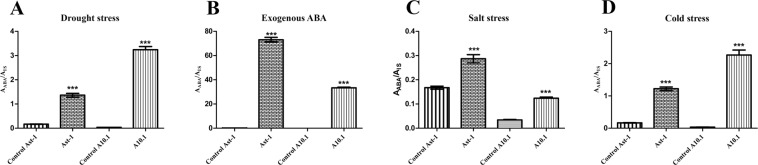
LC-MS analysis of leaf ABA accumulation in A10.1 and Ast-1 accessions of *Setaria viridis* submitted to (**A**) drought, (**B**) exogenous ABA, (**C**) salt and (**D**) cold. The results are shown as the peak area ratio from samples and peak areas from the internal standard (A_ABA_/A_IS_). Asterisks indicate statistically significant differences *t*-test (*P* < 0.001).

An increase in ABA levels was detected in leaves of Ast-1 and A10.1 plants after all treatments, as expected. The results were expressed as the ratio of the peak areas obtained for the samples (A_ABA_) and peak areas from internal standard (A_IS_), as shown in Fig. 7. During drought and cold stresses, A10.1 plants showed an increase of ~3-fold in leaf ABA levels, with the double observed for Ast-1 (Fig. 7A,D). In salt stress conditions, these results were inverted, with Ast-1 plants having a 2-fold increase in ABA levels higher than A10.1 plants (Fig. 7C). In plants treated with exogenous ABA, the levels of ABA accumulation drastically increased when compared to non-treated plants (Fig. 7B).

## Discussion

The core ABA signaling components are promising targets for plant genetic engineering towards improving important agricultural traits such as plant biomass, yield and tolerance to abiotic stresses, as this phytohormone is involved in many aspects of plant growth, development and responses to environmental changes. Some monocot, C4 plants including maize, sorghum, switchgrass and sugarcane are important crops for human and animal feeding, in addition to their use as biofuels feedstock^34^. In general, genetic transformation of these plants is laborious and time-consuming, in addition to genome sequences not fully annotated, making functional genomics studies more difficult. Thus, the use of model plants with fast growth and short life cycles, suitable transformation protocols and genome fully available is pivotal to accelerate genomic studies and, if possible, translate them to target crops. In this context, *S*. *viridis* emerged as a powerful model for C4 plants, as demonstrated by a diverse array of studies^35^. In order to better characterize this model plant, the main objectives of the present work were to identify, characterize and perform a detailed analysis of the gene expression pattern of its core ABA signaling system that includes the pyrabactin-like receptors (PYL), Ser/Thr phosphatases (clade A PP2Cs) and *Sn*f1-related protein kinases (SnRK2s). In this study, two different accessions of *S*. *viridis*, (A10.1 and Ast-1) were used and submitted to drought, salt and cold stresses, in addition to exogenously applied ABA, in order to analyze possible differences in the gene expression of this core ABA signaling system within the species.

Eight putative *SvPYL* genes were found in the *S*. *viridis* genome. These genes encode proteins containing the polyketide cyclase 2 domain (PF10604), which is a subfamily of Bet v 1-like superfamily, characterized by presence of a hydrophobic cavity that acts as ABA binding site^4^. Our analysis revealed that *S*. *viridis* has the same number of *SvPYL* genes found in sorghum^12^, but a lower number compared to maize, which contains 11 PYL genes^13^, and to the more distant *A*. *thaliana*, which has 14 PYL receptors genes^15,16^. Besides the conserved domain PF10604, plant PYL proteins characterized up to now are known to have the ‘GATE’ and the ‘LATCH’ conserved domains, which are β sheet loops present in all PYL protein sequences. The results presented here also demonstrated that the eight putative SvPYL proteins also contain these domains. The binding of ABA lead to conformational changes of these ‘GATE’ and ‘LATCH’ domains, which in turn facilitate ABA-mediated interaction of PYL with the protein phosphatases PP2Cs^36^. In *Arabidopsis*, PP2C genes are categorized into 13 subfamilies, from A to L^37,38^, where the clade A subfamily consisting of 9 proteins, contains 6 PP2Cs that act as negative regulators of ABA signaling^4^. Based on the presence of highly conserved amino acid residues involved in PYL and SnRK2 protein interaction, in the binding of the cofactors Mn^2+^/Mg^2+^ and constituting the domain PF00481, we found 12 putative PP2C-encoding genes in *S*. *viridis* genome, all of them clustering in the clade A of other plant species PP2Cs^12,39,40^. However, in 4 out of 12 SvPP2Cs proteins (SvPP2Cs2.2/2.3, 3.2 and 11), the amino acid residues involved in PYL and SnRK2 interactions were not found, suggesting that these proteins are not functional. The high orthology of SvPP2Cs with other plant PP2C deduced from the phylogenetic analyses indicate their close evolutionary relationship.

The modulation of signal transduction pathways is often controlled by reversible phosphorylation of proteins. In this regard, the subclass III plant-specific sucrose non-fermenting 1-related subfamily 2 (SnRK2) protein kinases have been implicated in ABA signaling as important modulators^4^. In *Setaria*, eleven putative SnRK2 genes were identified. Based on the presence of conserved domains such as PF0069, ATP-binding loop, activation loop, PP2C interface residues, SnRK2 box and ABA box^12,40–42^. Similar number of *SnRK2* genes was also identified in other higher plants such as Arabidopsis, maize and sorghum, where 10 *SnRK2s* were found in each species^12,13^. Since all the genes coding for the core factors of ABA signaling system were also identified in *S*. *viridis*, this system should function as described for other plants, with binding of PYL proteins to PP2Cs (and therefore inhibiting these phosphatases) in the presence of ABA, which in turn allows accumulation of phosphorylated SnRK2s responsible for subsequent phosphorylation of ABA-responsive element binding factors^43^ (ABFs). The ABFs are responsible for the activation of ABA-related genes, which control different aspects of plant growth, development and responses to environmental changes.

To gain insight on global gene expression of identified ABA core components in *Setaria* under abiotic stresses, qRT-PCR experiments were performed using RNA extracted from leaves of A10.1 and Ast-1 accessions of *S*. *viridis* submitted to different abiotic treatments. The time points chosen for gene expression analysis were based on physiological measurements, particularly to select plants showing decreased rates of photosynthesis after stress application. The expression profiles of most of the genes coding for the core ABA signaling components were quite variable throughout the time between the different treatments and between the two *S*. *viridis* accessions. Our data was presented as fold change in gene expression in relation to the beginning of the experiments (time 0 h), and it is worth to note that even in control conditions most of the core ABA signaling components have variable expression levels throughout time. This may indicate that *PYLs*, *SnRK2s* and *PP2Cs* regulate their expression levels to maintain plant homeostasis even when plants are not submitted to drastic environmental changes. As the application of ABA and cold treatment did not drastically changed the expression profile of *S*. *viridis PYL*, *PP2C* and *SnRK2* genes, the discussion will be focused on drought and salt stresses. However, physiological responses (Fig. 5) and accumulation of endogenous ABA levels (Fig. 7) were observed in all conditions tested, and the gene expression profile obtained is probably reflecting our specific experimental conditions.

### Regulation of *SvPYL* genes

In all conditions tested, expression levels of the *SvPYL* genes may be considered low or downregulated, compared to those of *SnRK2* and *PP2C* genes. In some species such as *A*. *thaliana* and *Zea mays*, *PYR/PYL/RCAR* genes are usually expressed constitutively or immediately after the perception of stress signals to sense changes in ABA^4,13,43,44^. In this work, expression analysis was performed based on decreased rate of photosynthesis after the stress application and, possibly, the stresses were perceived before any physiological penalties could be observed, partially explaining the low levels or the downregulation of *SvPYL* gene expression determined in our experimental conditions. The most distinctive results from *PYL* gene expression analysis were noticed in salinity conditions, where the majority of the genes were downregulated in A10.1 plants in contrast to Ast-1 genotype, which did not show decrease in most of the *PYL* transcript levels (Supplementary Fig. S4–7). These results were corroborated after HCA and PCA studies, which demonstrated low variance levels of the *SvPYL*s in the loading of PCA (Supplementary Fig. 8C), indicating that this family, in our experimental conditions, does not have influence in the multivariate analysis, independent of the stress applied. In fact, we observed that the exclusion of *SvPYL*s from HCA and PCA analysis changed the profile of the clustering (Supplementary Fig. 8), creating a new group (shown in blue in Supplementary Fig. 8D).

### Regulation of *SnRK2* genes

The *SnRK2* kinase genes were differentially expressed in *S*. *viridis*, depending on the accession and the stress applied (Supplementary Fig. S4–7). Similar results were obtained when expression patterns of wheat *SnRK2* genes were analyzed under similar conditions, with ABA application showing the weakest stress response among the treatments^45^. It was observed that *SvSnRK2*.*4*, *SvSnRK2*.*9*, *SvSnRK2*.*10* and *SvSnRK2*.*11* were the genes most up-regulated under drought and salt stresses in *S*. *viridis*, especially in Ast-1 accession. The protein orthology analysis revealed that SvSnRK2.4 and SvSnRK2.11 have similarity with AtSnRK2.7/AtSnRK2.8 and AtSnRK2.9, respectively, while SvSnRK2.9 and SvSnRK2.10 showed no orthology with other *Arabidopsis* kinases (Supplementary Table S1). However, SvSnRK2.9 and SvSnRK2.10 have orthology with the SbSnRK2.9 and SbSnRK2.10 kinases identified in sorghum^12^. The HCA and PCA studies corroborated the results described above, clearly showing that *SnRKs 2*.*9* and *2*.*11* genes were highly expressed in *S*. *viridis* during drought and salt stress episodes (Figs 6A and S4-S5). The most responsive *SvSnRK2* genes to drought and salt stresses do not belong to the subclass III (*SvSnRK2*.*1*, *SvSnRK2*.*2* and *SvSnRK2*.*3*), but to the subclasses I (*SvSnRK2*.*10* and *SvSnRK2*.*11*) and II (*SvSnRK2*.*4* and *SvSnRK2*.*9*). SnRK2 members of the subclass III are known to be strongly activated by ABA, acting as positive regulators of ABA signaling^18,42,46,47^. However, in agreement with our analysis in *S*. *viridis*, *SnRK2* genes from subclasses I and II were upregulated in the phylogenetically related plant sugarcane (*Saccharum officinarum*) submitted to NaCl and PEG treatments^48^. In addition, subclass II SnRK2s 2.7 and 2.8 from Arabidopsis, which have high orthology with SvSnRK2.4, have been demonstrated to participate in the regulation of some drought-responsive genes involving the ABA-responsive element binding factors AREB/ABF^49^. More recently, protein-protein interactions studies revealed that homo- and heteromerization of OST1 (subclass III AtSnRK2.6) with AtSnRKs 2.2, 2.3 and 2.8 occurred during osmotic stress in Arabidopsis. In addition, several OST1-complexed proteins were identified as type 2A protein phosphatase (PP2A) subunits, suggesting that broad interaction network between SnRK2-type protein kinases and PP2A-type protein phosphatases other than the well-established interactions of SnRK2-type protein kinases with PP2Cs can occur^50^. The high responsiveness of subclasses I and II *SvSnRK2* genes in our experimental conditions reinforces the results described above, especially because these SvSnRK2 genes were strongly downregulated after plant rewatering, suggesting the involvement of SvSnRKs 2.4, 2.9, 2.10 and 2.11 in drought responses of *S*. *viridis*.

### Regulation of *SvPP2C* genes

The group-A phosphatase *PP2C* genes were largely activated in drought conditions in both *S*. *viridis* accessions and in A10.1 plants under salt stress (Figs 6A and S4-S5), and these results were clearly observed after PCA analysis (Fig. 6B,C). Among the most expressed *PP2C* genes in *S*. *viridis* under salt and drought stresses are *PP2Cs 3*.*2*, 5, 6, *7*.1 and *7*.*2*.

The PP2Cs, in addition to SnRK2s proteins, can interact with transcription factors and other phosphatases and kinases, altering their activities in response to abiotic stresses. In this regard, it has been reported that AtPP2Cs interact with kinases such as SnRK3/CIPK and the mitogen-activated kinase kinase kinase δ4 (MAP3K δ4). Ohta *et al*.^51^ demonstrated that two Arabidopsis group A PP2Cs, ABI1 and ABI2, interact with several SnRK3/CIPKs, which are members of the SnRK3/calcineurin B-like (CBL)-interacting protein kinase (CIPK) families, responsible for mediating various signaling pathways through interactions with CBL proteins. Moreover, it has been shown that SnRK3.17/CIPK3 is involved in the induction of gene expression in response to ABA, cold and high salinity^52^, indicating that these proteins might be involved in PP2C-mediated ABA signaling. Therefore, group-A PP2Cs might form a complex signaling network not only with SnRK2, but with a myriad of proteins such as kinases, phosphatases, transcription factors and metabolic enzymes, to fine-tune ABA signaling in plants under abiotic stresses^43^. This fine-tuning is extremely important to balance the positive effects of ABA on plant survival during stress and the detrimental effects of this hormone on plant development and growth. Our results on gene expression analysis show that PP2Cs are the most responsive components of the core ABA-signaling network in *S*. *viridis* under abiotic stresses, corroborating the importance of PP2Cs to the fine-tuning of the complex ABA responses in monocot plants.

Importantly, our studies performed using the gene expression profile of the core ABA signalling components showed that the expression of these genes decreases in a time-dependent manner, suggesting that rapid ABA responses are achieved under abiotic stresses in *S*. *viridis*. Thus, a careful experimental approach should be taken into consideration when studying ABA signalling using this experimental model. In addition, it was observed clear differences in gene expression of ABA signalling core components between the two accessions studied. The accession A10.1 appeared to be more responsive to osmotic stresses when compared to genotype Ast-1 (Fig. 6B,C).

Based on the results described above, current studies are being performed by our group using protein-protein interaction analysis and transgenic *S*. *viridis* A10.1 to demonstrate this complex ABA signaling network involved in abiotic stress responses in monocot plants. We are using the most expressed targets during our experimental conditions to corroborate the gene expression studies.

### Physiological responses of *S*. *viridis* genotypes under different abiotic stress treatments

In addition to the differential gene expression patterns of the core ABA signaling network components observed for A10.1 and Ast-1 accessions, the results also demonstrated that different accessions have distinctive physiological responses under abiotic stresses (Fig. 5). The different physiological responses observed for A10.1 and Ast-1 accessions under drought stress were already reported in a study by Saha *et al*.^28^ and, as discussed by the authors, these differential responses are probably due to distinctive osmotic adjustments that ultimately lead to changes in the root and leaf radial water movement^28,53^. As radial water movement through the xylem can be determined by anatomical and morphological structures, the different physiological responses between the accessions is not surprising, since A10.1 and Ast-1 have distinguishing morphology (Supplementary Fig. S2). Obviously, other factors such as cellular and molecular components might be involved in the physiological responses during osmotic stresses in plants, for instance aquaporins membrane water channels, chaperones and osmolytes^11,53^. Moreover, the differences in gene expression and physiological traits in response to abiotic stresses observed between A10.1 and Ast-1 accessions may reflect the different geographical locations from where these accessions originated, since the environmental factors by which these accessions are submitted are also different. Thus, the distinctive responses of A10.1 and Ast-1 plants reflect the diverse adaptive mechanisms that allow plants to survive under adverse environmental conditions.

In summary, the present study allowed a detailed analysis of gene regulation of the core ABA signaling components in *Setaria viridis* submitted to different treatments and provided suitable targets for genetic engineering of C4 plants for tolerance to abiotic stresses.

## Materials and Methods

### Identification of ABA receptor PYR/PYL/PP2C/SnRK2 in *Setaria viridis*

Two strategies were used to identify the core ABA components in the genome of *Setaria viridis*. In the first strategy, a total of 2,819, 17,744 and 1,586 protein sequences corresponding to PYR/PYL, PP2C protein phosphatases and SnRK2s, respectively, available in the National Center for Biotechnology (NCBI), were downloaded. Tblastn searches were performed using an e-value cutoff set to 10^−10^ against genome files of *S*. *viridis* v1.1 downloaded from Phytozome v12.1 database (phytozome.jgi.doe.gov/pz/portal.html). The redundant sequences were removed using a custom Perl. After removal of the redundant sequences, putative sequences were screened for the existence of domains by Pfam^54^. The specific domain observed was the polyketide cyclase2 domain (PF10604) to PYR/PYL, PF00481 to PP2Cs and SnRK-specific Pfam domain (PF0069). Protein sequences with no catalytic domain were excluded from the dataset. Molecular weight (MW), theoretical isoelectric point (pI) and protein length (aa) were manually calculated using the EXPASy server (web.expasy.org/protparam/). Position of domain was predicted in HMMSCAN (www.ebi.ac.uk/Tools/hmmer/search/hmmscan).

The second strategy searched *PYR/PYL/RCAR*, *PP2C* and *SnRK2* genes from *S*. *viridis* and their orthologs in *Arabidopsis thaliana*, *Oryza sativa*, *Sorghum bicolor* in the GreenPhyl plataform (www.greenphyl.org/) using the keywords PYL/PYR/RCAR, PP2C and SnRK2. We identified a total of 35, 45 and 54 sequences for *PYL/PYR/RCAR*, *PP2C* and *SnRK2*, respectively. BLASTn searches were carried out using these sequences as query against the *S*. *viridis* genome (e-value < e^−10^) to isolate *S*. *viridis* genes that were further translated to compare their corresponding polypeptides with those of other species using the MAFFT program^55^, available on the Galaxy instance^56^ of the South Green Platform (www.southgreen.fr/).

The conserved amino acids were identified using the GeneDoc program (www.nrbsc.org/old/gfx/genedoc/). Genes that did not contain specific domains were removed. To perform phylogenetic analyses, the families were extended by searching for homolog proteins in complete proteomes of interest (i.e. *S*. *viridis* and *A*. *thaliana*). Firstly, a Hidden Markov Model (HMM) profile was built using hmmbuild of the HMMER software package^57^ from the core polypeptides of the family, aligned with the MAFFT program^55^, using a maximum of 100 iterations, and cleaned with GBlocks^58^. Secondly, the complete proteome was screened with this profile using hmmsearch to extract the family sequences.

### Phylogenetic, exon/intron and motif analysis

For phylogenetic analysis, amino acid sequences of putative PYL, PP2C and SnRK2 proteins of *S*. *viridis*, *S*. *bicolor*, *O*. *sativa* and *A*. *thaliana* were analyzed. The genes of *S*. *viridis* were named based on numbering of *S*. *bicolor* and *A*. *thaliana* orthologs genes, estimated in Proteinortho^59^ v5.11 with an algebraic connectivity threshold of 0.1 and e-value of 10^−5^. Sequences were aligned with MUSCLE program and phylogenetic trees were constructed using FastTree 2.1.5 program^60^. The trees were visualized using the online program ITOL (itol.embl.de/). The sequences data of *SvPYL*, *SvPP2C* and *SvSnRK2* genes were deposited in the GenBank database under the mnemonic numbers MG766907 to MG766942. The exon/intron structure of these genes was examined using Gene Structure Display Server (GSDS, gsds.cbi.pku.edu.ch). Pfam prediction and conserved evolutionary domains were analyzed using Multiple EM for Motif Elicitation (MEME Suite 4.11.1) server software^61^.

### Promoter analysis

From the transcription start codon 1500 bp was used as promotor sequences of ABA signaling core components were retrieved using a custom script in Perl language. The analysis to identify cis-acting regulatory elements (CARE) ABRE, LTRE and MBS in the upstream DNA sequence was performed using PlantCARE program^62^ (bioinformatics.psb.ugent.be/webtools/plantcare/html/).

### Plant material

Seeds of *S*. *viridis* accessions A10.1 and Ast-1 were germinated in pots containing soil, substrate (Plantmax) and vermiculite (Agrifloc, Brasil Minérios) in a mixture of 3:1:0.5 (w/w/w). Plants were maintained in a growth chamber (Conviron PGW40) under 16 h photoperiod at 450 μmoL m^−2^s^−1^ light intensity, 25 ± 2 °C and 65% relative humidity.

### Abiotic stress treatments and physiological measurements

Before treatment, plants were grown under the conditions described above until the reproduction phase^63^ (RP; 32 days after planting DAP). Plants were watered to field capacity every day and fertilized once a week with a solution of 2.5 g.L^−1^ Plantafol N:P:K (20:20:20). Physiological measurements were performed in the flag leaf using an open gas exchange system with a 6 cm^2^ clamp-on leaf cuvette (LI-6400XT, LICOR). Photosynthetic photon flux density (PPFD) was fixed at 1,500 μmoL m^−2^s^−1^ and 400 ppm CO_2_ using the built in red-blue LED light of the leaf cuvette.

### Drought stress treatment

Water-deficit stress (WS) was applied in both accessions by withholding the water supply to pots containing 32 DPG plants for 45 h. The net photosynthesis rate was monitored during WS until a decay in the photosynthesis rate was observed. After the period of WS, plants were re-watered keeping the net photosynthesis rate monitored. A well-watered (WW) set of plants at the field capacity for both accessions were grown under the same conditions with similar irrigation and fertilization regimes as described before. Leaf samples were collected from water-stressed, re-watered and control plants, frozen in liquid nitrogen, and stored at −80 °C for further analysis.

### Salt stress treatment

For the salt treatment, different concentrations of NaCl were used for both A10.1 and Ast-1 accessions. The NaCl concentrations used were 0, 50, 100 and 200 mM. The plants were irrigated with salt solution every day until field capacity. Photosynthesis was measured every 24 h until 96 h after the application of NaCl solution. Leaf samples were collected during the treatment in 0, 24, 48, 72 and 96 h, frozen in liquid nitrogen and stored at −80 °C for further analysis.

### Exogenous ABA treatment

In the exogenous ABA application assay, plants in the RP were sprayed on the leaves with 0 (control), 50 and 100 μM of ABA [(±) ABA, Sigma] for A10.1 accession and 0, 100 and 200 μM exogenous ABA for Ast-1 for 6 h. The net photosynthetic rate was measured at 2 h intervals until 6 hours after the application of ABA solution. Leaves were collected after 0, 2, 4 and 6 h treatment, frozen in liquid nitrogen and stored at −80 °C for further analysis.

### Cold stress treatment

For imposing the cold stress, the plants were submitted to decay of 5 °C per day in temperature. The initial temperature was 25 °C until the final temperature reached 5 °C. Photosynthesis analysis was performed every 24 h in different temperatures (25, 20, 15, 10 and 5 °C). Plants of the control group were kept at 25 °C. Leaf samples were collected in all temperatures, frozen in liquid nitrogen and stored at −80 °C for further analysis.

### RNA isolation and cDNA synthesis

Total RNA was isolated from 200 mg of leaf samples using TRIzol Reagent (Thermo Scientific), according to the manufacturer’s instructions. Genomic DNA was removed using RQ1 RNase-free DNase (Promega), according to the manufacturer’s instructions. Total RNA was quantified using a NanoDrop ND-1000 Spectrophotometer (Uniscience), and RNA orintegrity was verified in agarose gel electrophoresis. Reverse transcription reaction was carried out with 1 μg of total RNA and oligo (dT) in a total volume of 20 μL using RevertAid First Strand cDNA Synthesis Kit (Thermo Scientific), following the manufacturer’s recommendations. cDNA samples were diluted (1:20) prior to use in RT-qPCR assays.

### qRT-PCR analysis

Primers of ABA receptors were designed using the software PrimerQuest (IDT). RT-qPCR was carried out in a 96-well optical plate with a StepOnePlus Real-Time PCR Systems (Applied Biosystems). Reactions were performed using Platinum SYBR Green PCR SuperMix-UDG with ROX (Invitrogen), 0.2 μM of each primer and 1 μL of diluted cDNA (1:20) in a final volume of 10 μL. The following thermal cycling condition was used for all amplifications: 2 min at 50 °C min, 20 sec at 95 °C, followed by 40 amplification cycles of 95 °C for 3 sec, and 60 °C for 30 sec. After 40 cycles, the melting curve and amplification curve were checked to evaluate specific amplification. The relative expression levels of these genes were analyzed by the 2^−∆∆Ct^ described in Schmittgen & Livak^64^, using the cullin (*SvCUL*), clathrin adaptor complex (*SvCAC*), translation factor SUI1 (*SvSUI*), eukaryotic initiation factor 4- alpha (*SvelF4α*) and elongation Factor 1-alpha (*SveF1α*) genes as the internal controls^63^. The amplified primers and internal controls were listed in supplementary materials (Table S6).

### ABA extraction

Leaf samples from all treated and control plants in the final set point of analysis were collected and freeze-dried. Samples were ground and 150 mg of powdered materials supplemented 50 μL of 3,5-Dichloro-4-hydroxybenzoic acid 0.1 g.L^−1^ (Sigma) was added (internal standard; IS) and extracted at 4 °C for 12 h under agitation in 10 mL of 80% methanol. The solution was centrifuged at 4 °C and 10.000 *g* for 5 min. The supernatant was evaporated, and the aqueous residue was adjusted to 5 ml with water before passed through preconditioned C18 SPE cartridges (3 mL, 500 mg), with 3 mL deionized water, followed by 3 mL methanol. The cartridges were washed with 1 mL 20% methanol containing 0.1% (v/v) formic acid and the retained phytohormones were eluted with 1 mL 80% methanol. The extract was evaporated in vacuum at room temperature and adjusted for 300 μL with water.

### ABA detection by HPLC-(ESI)-MS

Leaf samples from both treated and control plants were analyzed by high performance liquid chromatography coupled to mass spectrometry (LC-MS) for determination of ABA accumulation. The samples were analyzed in a Shimadzu Nexera XR, equipped with LC-20AD- XR pumps, coupled to a Bruker Daltonics Amazon SL mass spectrometer with an Ion Trap analyzer and the data acquisition were carried out with the Compass Data Analysis data system (Bruker Daltonics). Chromatographic analysis of the leaf extracts for determination of ABA proportion was performed on an Agilent Eclipse Plus C18 RRHD analytical column 2.1 mm internal diameter × 50 mm length, 1.8 μM particle size placed in an oven maintained at 40 °C using 0.1% formic acid as solvent A and methanol plus 0.1% formic acid as solvent B with the following gradient elution program: at 0 min, it started with 20% B which was increased to 50% B in 8 min, then to 100% B in 8.5 min, continued at 100% B to 12 min for washing and was equilibrated back to 20% B from 12.1 min to 15 min at a solvent flow rate of 0.3 mL/min. Electrospray ionization method was used for mass spectrometry under the following conditions: spray voltage (negative mode = 4000 V); temperature of the capillary 180 °C. The MRM (Multiple Reaction Monitoring) mode was used for determination of ABA and IS. ABA and IS were monitored at m/z transitions of 263 → 153, 219; 204.6 → 160.7, respectively. The normalized collision energies for ABA and IS were 0.30 and 0.35, respectively.

### Statistical analyses

Experimental data were analyzed using randomized block design (RBD) with replications for each treatment (drought, salt, cold and exogenous ABA). Differences among treatments per sample were analyzed using unpaired t-test on GraphPad Prism software (GraphPad Software Inc., La Jolla, CA, USA).

### Multivariate Analysis

The data of gene expression were submitted to multivariate analysis by unsupervised methods (Principal Component Analysis – PCA; Hierarchical Clustering Analysis – HCA) to determine the relationship between the different levels of expression of the analyzed genes and the different experimental conditions which the individuals of two accessions of *S*. *viridis* (A10.1 and Ast-1) were submitted.

As the first step, the data related to the levels of gene expression during all four types of stress applied in the plants (salinity, drought, cold and exogenous ABA) were organized in a single spreadsheet and subsequently submitted to a pre-treatment. First, all data was log transformed by the following equation: log 10 (x + 1), where x is the level of gene expression for each gene in each experimental condition observed, and then mean centred.

The pre-treated data were used to perform HCA analysis by Ward’s^65^ method 1 using the software Origin^®^ Pro version 9 and a PCA analysis with bootstrap (N = 100). In addition, the data was used to create a heat-map using the software PAST 3.19, to establish the relationship between gene expression profile, stress treatments and plant accession^66–69^.

## Supplementary information

Supplementary information
