# Supplementary material for: Identification and characterization of core abscisic acid (ABA) signaling components and their gene expression profile in response to abiotic stresses in Setaria viridis
Source: Sci Rep. 2019 Mar 11;9:4028. doi: 10.1038/s41598-019-40623-5 (PMC6411973; doi:10.1038/s41598-019-40623-5)
Supplement: Supplementary file 1 — Supplementary information [file 41598_2019_40623_MOESM1_ESM.pdf]

Identification and characterization of core abscisic acid (ABA) signaling components and their gene expression profile in response to abiotic stresses in *Setaria viridis*

Karoline Estefani Duarte[1,2], Wagner Rodrigo de Souza[2,3], Thaís Ribeiro Santiago[2], Bruno Leite Sampaio[2], Ana Paula Ribeiro[2], Michelle Guitton. Cotta[4], Bárbara Andrade Dias Brito da Cunha[2], Pierre Marraccini[1,5,6], Adilson Kenji Kobayashi[2], Hugo Bruno Correa Molinari[2*]

[1]Plant Biotechnology Program, Federal University of Lavras, Lavras, MG, 37200-000, Brazil.

[2]Genetics and Biotechnology Laboratory, Embrapa Agroenergy (CNPAE), Brasilia, DF, 70770-901, Brazil.

[3]Centro de Ciências Naturais e Humanas, Universidade Federal do ABC, São Bernardo do Campo, SP, 09606-045, Brazil.

[4]Department of Cell Biology, University of Brasilia, DF, 70910-900, Brazil.

[5]CIRAD, UMR AGAP (University Montpellier, CIRAD, IRD, INRA), Montpellier, 34398, France.

[6]CIRAD, UMR IPME (University Montpellier, CIRAD, IRD, Montpellier), Agricultural Genetics Institute, LMI RICE2, Hanoi, Vietnam.

[*]Corresponding author: Phone number: +55 61 3448 2307. E-mail address: hugo.molinari@embrapa.br

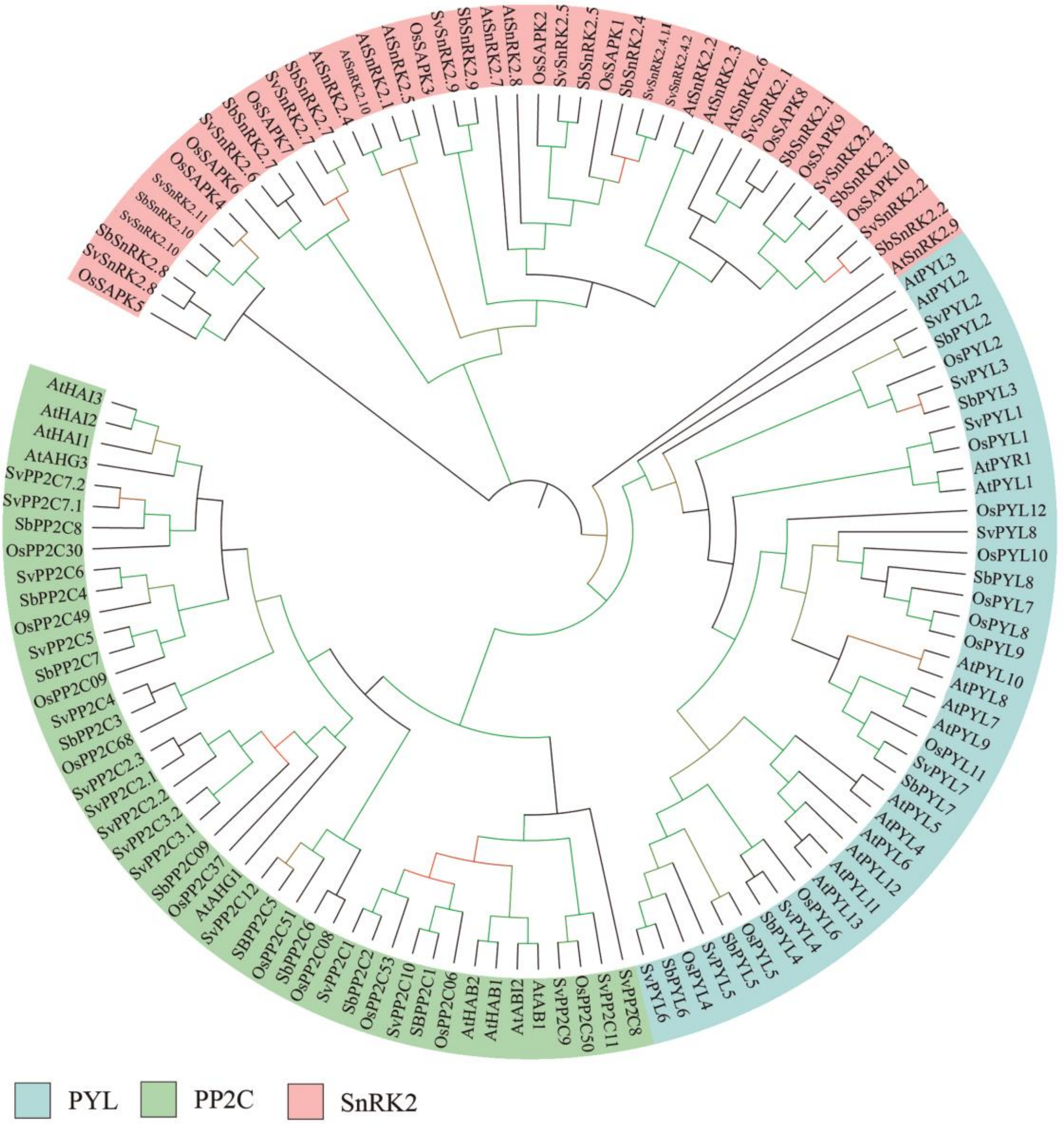

Figure S1. Phylogenetic analysis of ABA core signaling components from *Setaria viridis*. Maximum likelihood phylogeny of functionally characterized SvPYL / SvPP2C / SvSnRK2 proteins and their close homologs from *Arabidopsis thaliana* (At), *Sorghum bicolor* (Sb) and *Oryza sativa* (Os). The phylogenetic tree was constructed using FastTree 2.1.5 program. Branch color scale represents SH-like local support (red to lower values and green to higher values).

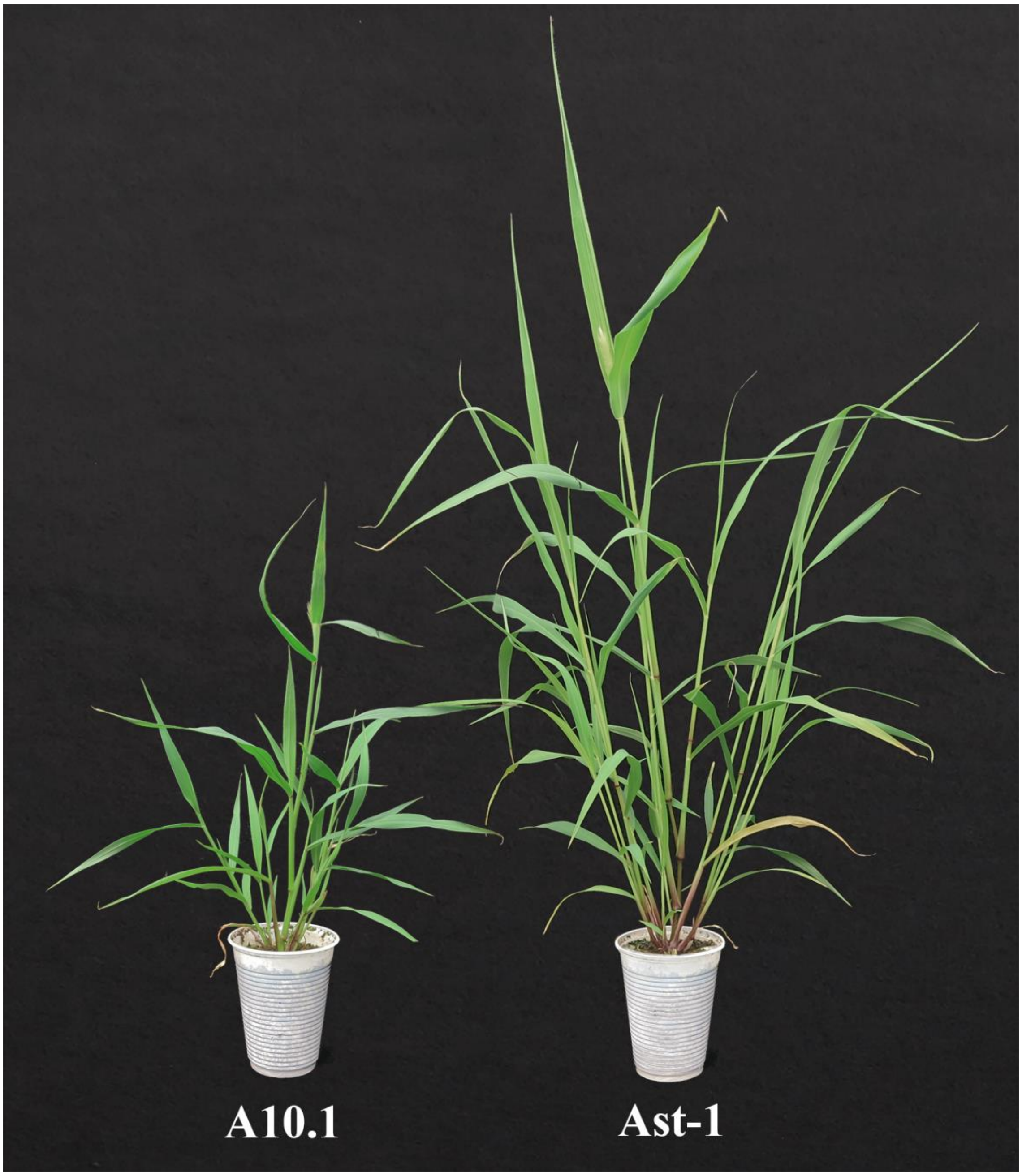

Figure S2. *Setaria viridis* accessions A10.1 (left) and Ast-1 (right) at the reproductive stage (RP), 32 days post-germination (DPG).

	Drought		Rewatering		Salt stress		Cold stress		Exogenous ABA	
Sv genes	A10.1	Ast-1	A10.1	Ast-1	A10.1	Ast-1	A10.1	Ast-1	A10.1	Ast-1
PYL 1	L	L			E					
PYL 2	E				E				L	
PYL 3	E	E			E	E				E
PYL 4	E	L			E	E			*	E
PYL 5					L	E				
PYL 6						E				
PYL 7	E	E								
PYL 8	E				E				E	
PP2C1	E	E								
PP2C2.1	L	L			E	L				
PP2C2.2		L								
PP2C3.1	E				E	E			L	L
PP2C3.2	E				E	L			E	E
PP2C4	E	E			E					
PP2C5	L	L			E				E	
PP2C6	E	L			E				E	E
PP2C7.1	E	L			E			*	E	E
PP2C7.2	E	L			E				E	E
PP2C8	L				L					
PP2C9	E	L			E				E	E
PP2C10	E	L			E	E			E	E
PP2C 12					E	L			E	E
SnRK 2.1		E								
SnRK 2.2	L									
SnRK 2.3	L	L			L				E	E
SnRK 2.4.1	L	L			E	E		*		
SnRK 2.4.2	L	L			E	E		*	E	
SnRK 2.5	E	L				E			E	
SnRK 2.6	E	E								
SnRK 2.7	E				E					
SnRK 2.8	E				E	E				
SnRK 2.9	E	L			E	L			E	E
SnRK 2.10	E	L			E					
SnRK 2.11	L	L			E	L				E

Down	Up	No change
2-3	2-3	p > 0.05
4-10	4-10	
>10	>10	

Figure S3. Expression profile of *SvPYL*, *SvPP2C* and *SvSnRK2* genes in different abiotic stresses in two accessions of *Setaria viridis* (A10.1 and Ast-1). The figure shows the fold-change in expression of genes related to the beginning of the experiment (0 h) compared to non-stress conditions (control). The fold-change is represented by the numbers in the squares located in the lower-left side of the figure. The asterisks represent $p > 0.05$ (non-statistically significant), unpaired t test. E and L conventionally corresponds to genes up- or down-regulated in early or late phases of the stress, respectively.

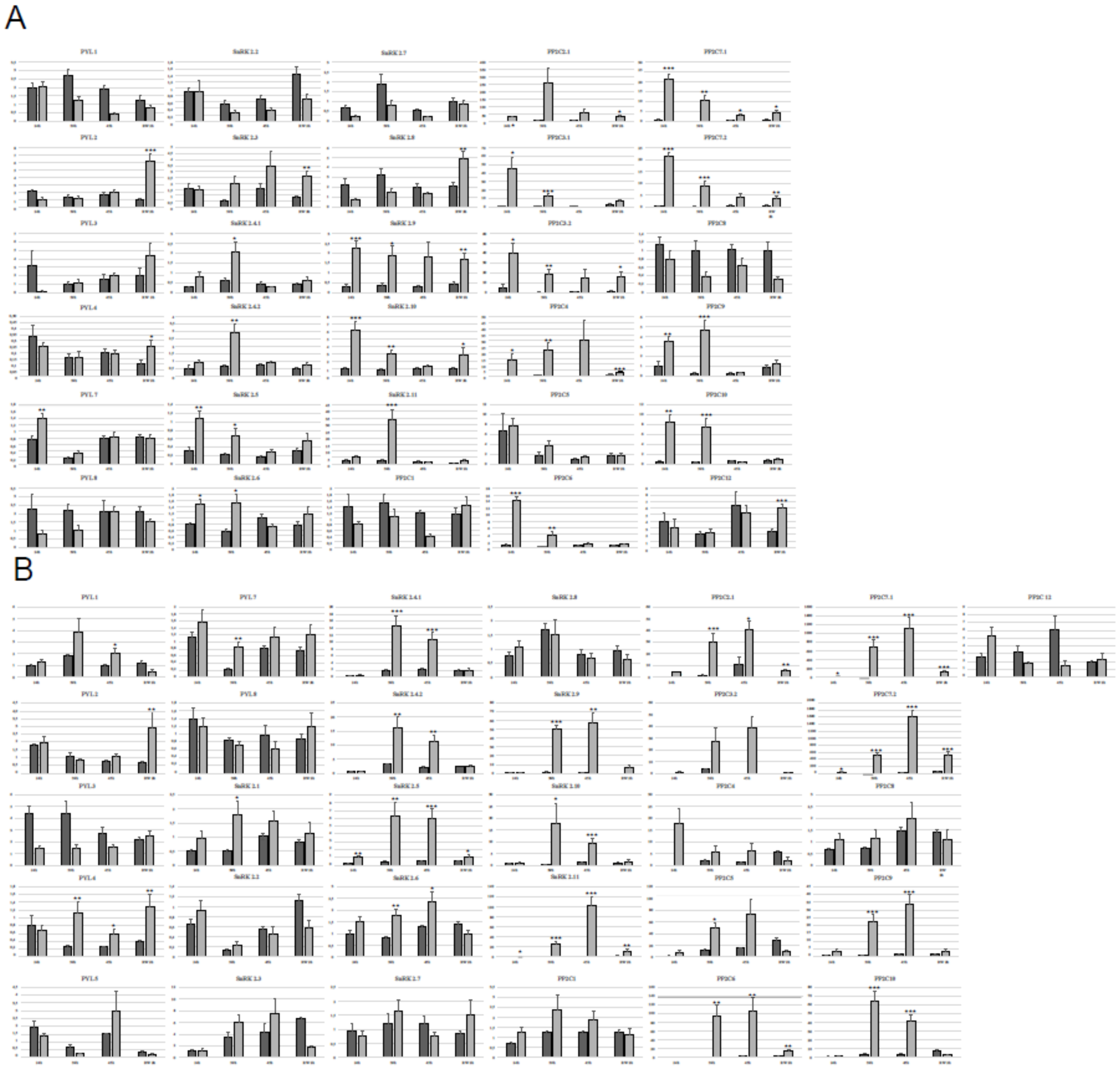

Figure S4. Expression profiles of *SvPYL*, *SvPP2C* and *SvSnRK2* genes in A10.1 (**A**) and Ast-1 (**B**) accessions of *Setaria viridis* submitted to drought stress. The figures show the fold-change in expression of genes related to the beginning of the experiment (0 h) compared to non-stress conditions (control). The asterisks represent (*$p > 0.05$; ** $p > 0.01$; ***$p > 0.001$) (non-statistically significant), unpaired t test.

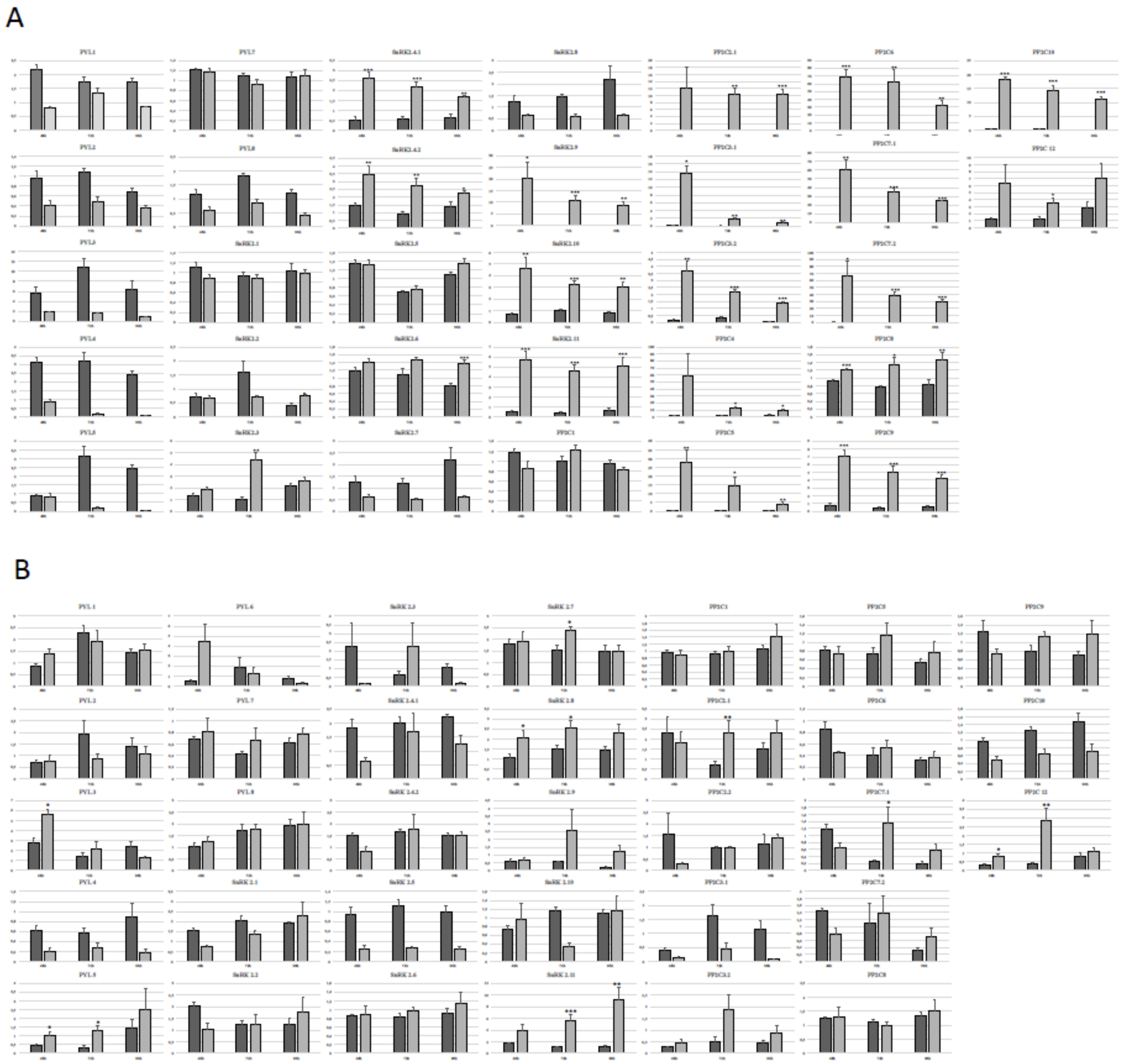

Figure S5. Expression profiles of *SvPYL*, *SvPP2C* and *SvSnRK2* genes in A10.1 (**A**) and Ast-1 (**B**) accessions of *Setaria viridis* submitted to salt stress. The figures show the fold-change in expression of genes related to the beginning of the experiment (0 h) compared to non-stress conditions (control). The asterisks represent (**p* > 0.05; ** *p* > 0.01; ****p* > 0.001) (non-statistically significant), unpaired t test

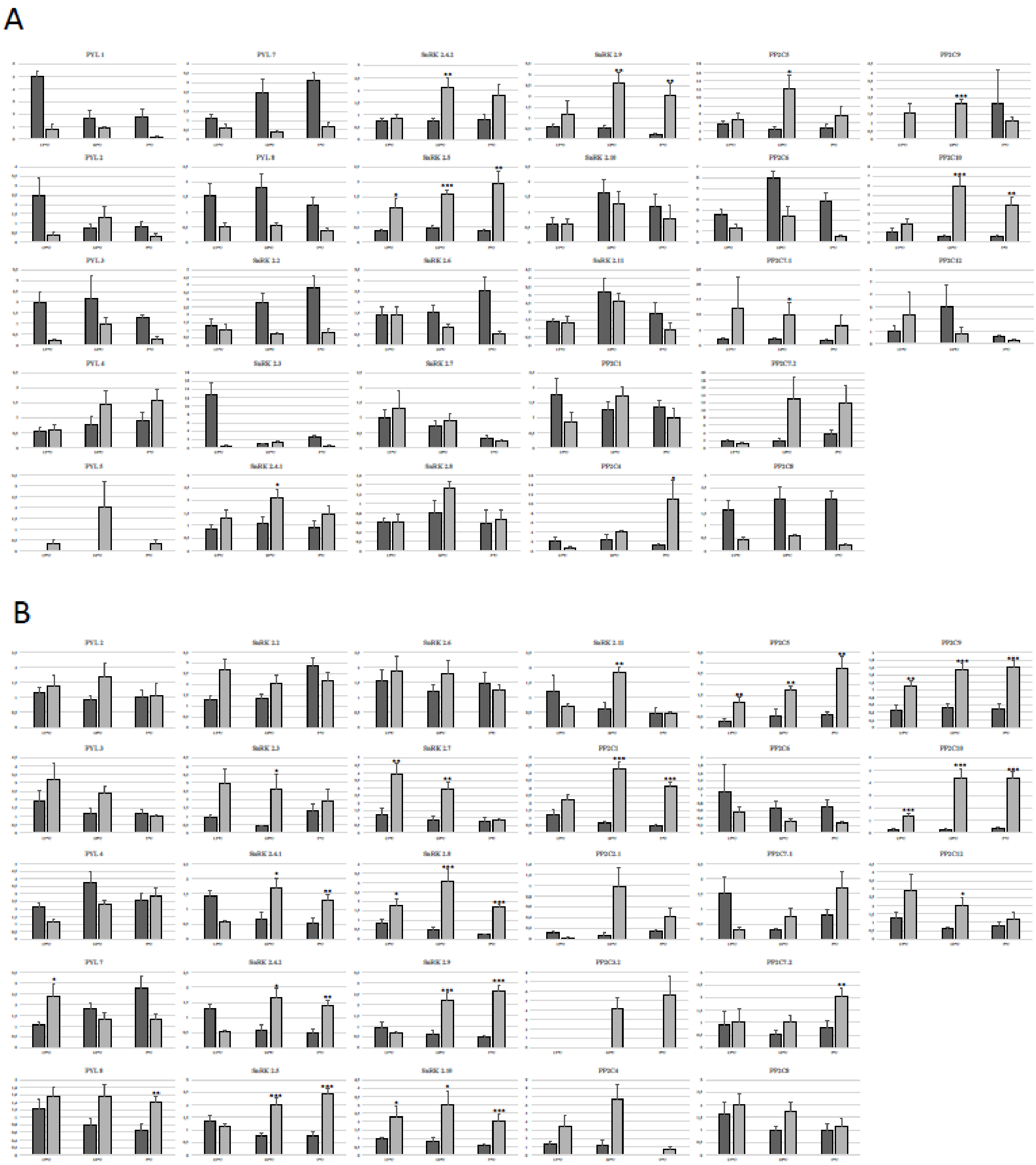

Figure S6. Expression profiles of *SvPYL*, *SvPP2C* and *SvSnRK2* genes in A10.1 (**A**) and Ast-1 (**B**) accessions of *Setaria viridis* submitted to cold stress. The figures show the fold-change in expression of genes related to the beginning of the experiment (0 h) compared to non-stress conditions (control). The asterisks represent (*p > 0.05; ** p > 0.01; ***p > 0.001) (non-statistically significant), unpaired t test

A

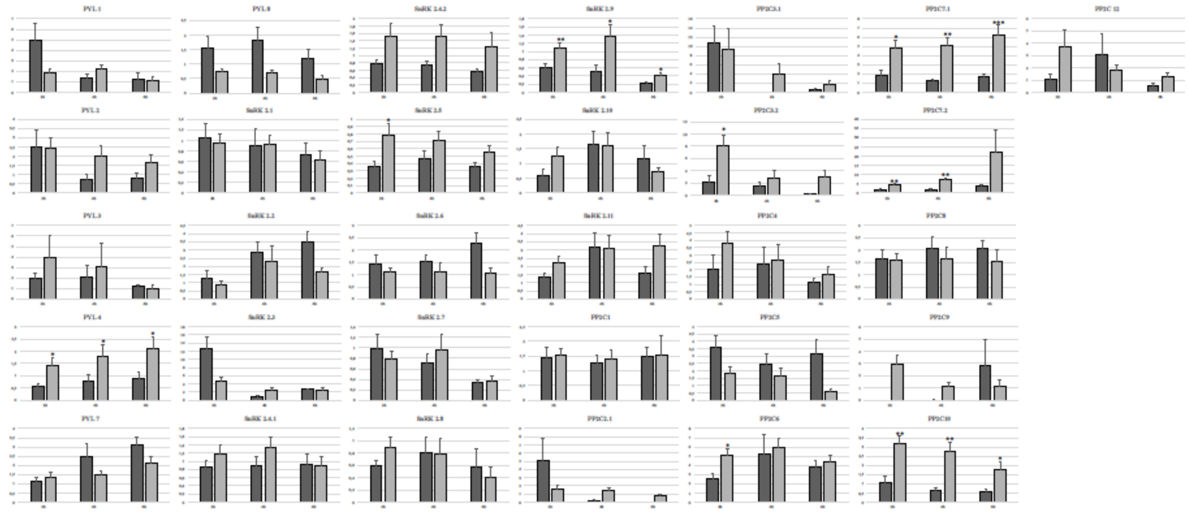

B

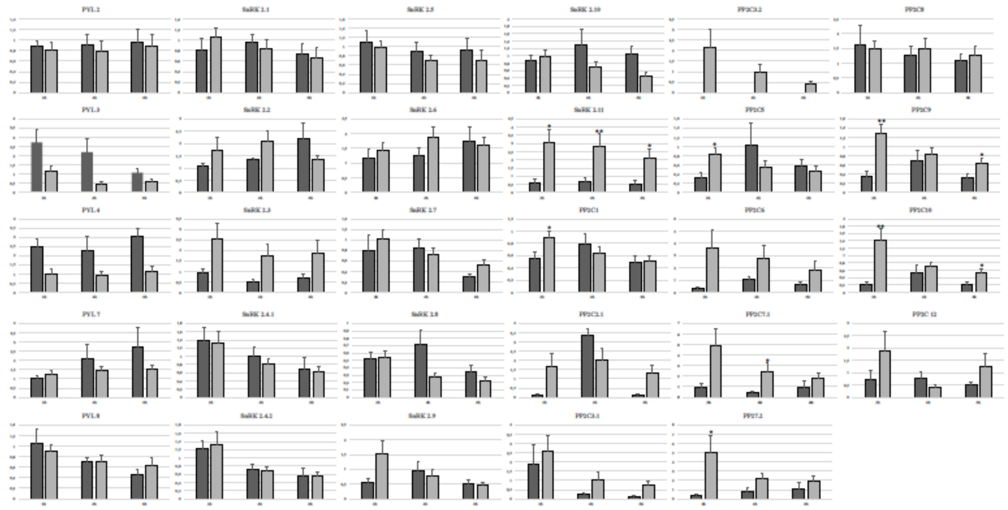

Figure S7. Expression profiles of *SvPYL*, *SvPP2C* and *SvSnRK2* genes in A10.1 (**A**) and Ast-1 (**B**) accessions of *Setaria viridis* treated with exogenous ABA. The figures show the fold-change in expression of genes related to the beginning of the experiment (0 h) compared to non-stress conditions (control). The asterisks represent (*$p > 0.05$; ** $p > 0.01$; ***$p > 0.001$) (non-statistically significant), unpaired t test

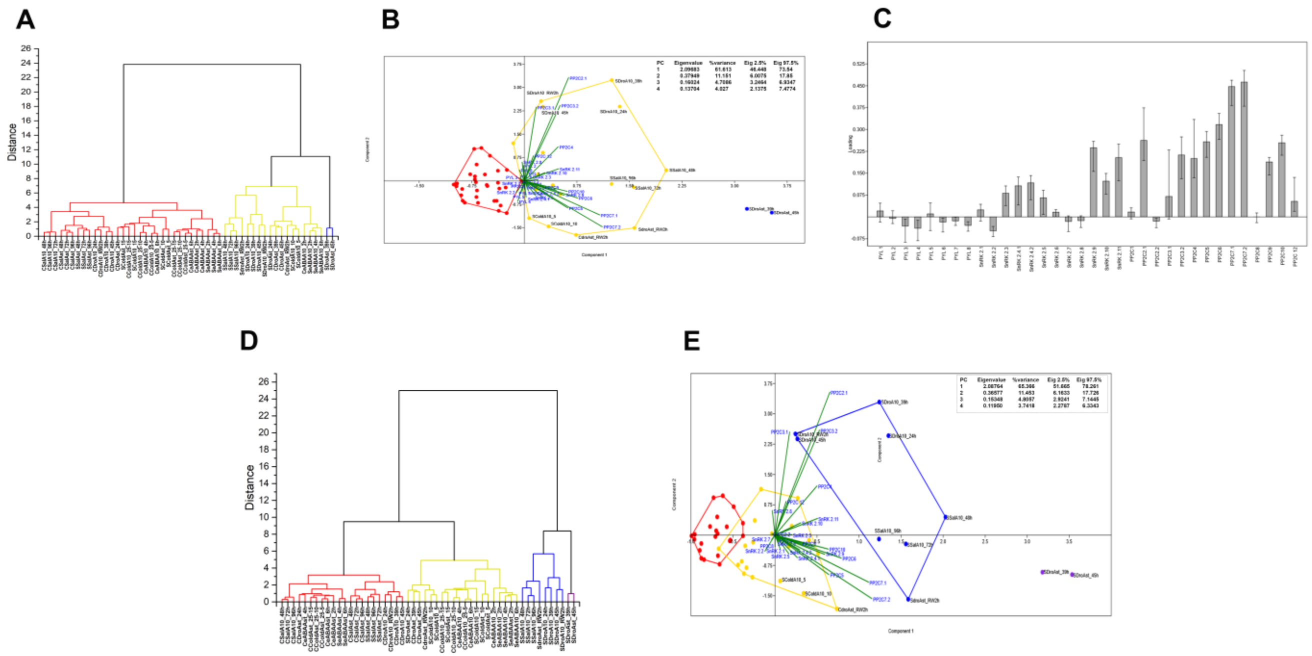

Figure S8. Multivariate analysis of expression profile of *SvPYL*, *SvPP2C* and *SvSnRK2* genes in different abiotic stresses in A10.1 and Ast-1 accessions of *Setaria viridis*. (**A**): Hierarchical cluster analysis (HCA) dendrogram for all treatments using the data for *SvPYL*, *SvPP2C* and *SvSnRK2*. Colours indicate grouping proposal. (**B**): Principal component analysis (PCA) exhibiting the correlation between all treatments and *SvPYL*, *SvPP2C* and *SvSnRK2* (**C**): Coefficient of loading using all the data from *SvPYL*, *SvPP2C* and *SvSnRK2*. (**D**): Hierarchical cluster analysis (HCA) dendrogram for all treatments using the data for *SvPP2C* and *SvSnRK2*. Colours indicate grouping proposal **(E)** Principal component analysis (PCA) exhibiting the correlation between all treatments (Drought, salt, cold and exogenous ABA) and *SvPP2C* and *SvSnRK2*. Legend: Control-C, Stressed-S, Drought-Dro, salt-Sal, cold-Col and exogenous ABA-eABA.

Table S1. Orthologs of SvPYL, SvPP2C and SvSnRK2 genes compared to those of *S. bicolor* and/or *O. sativa*, and *A. thaliana*.

# Species	Genes	Alg.-Conn.	Os_PYL	Sv_PYL	Sb_PYL	At_PYL
3	4	0.5	OsPYL1	SvPYL1	*	AtPYR1,AtPYL1
4	5	0.4	OsPYL10	SvPYL8	SbPYL8	AtPYL9,AtPYL8
3	3	1	OsPYL11	*	SbPYL7	AtPYL7
4	5	0.166	OsPYL2	SvPYL2	SbPYL3,SbPYL2	AtPYL2
4	4	0.5	OsPYL4	SvPYL6	SbPYL6	AtPYL6
4	4	0.5	OsPYL5	SvPYL5	SbPYL5	AtPYL5
4	4	1	OsPYL6	SvPYL4	SbPYL4	AtPYL4
2	2	1	*	SvPYL3	*	AtPYL3

# Species	Genes	Alg.-Conn.	Os_PP2C	Sv_PP2C	Sb_PP2C	At_PP2C
4	4	1	OsPP2C06	SvPP2C10	SbPP2C1	AtHAB1
3	5	0.166	OsPP2C08,OsPP2C51	SvPP2C12	SbPP2C6,SbPP2C5	*
4	9	0.14	OsPP2C09,OsPP2C30	SvPP2C7.2,SvPP2C7.1,SvPP2C5	SbPP2C8,SbPP2C7	AtHAI3,AtHAI2
4	5	0.166	OsPP2C37	SvPP2C3.1,SvPP2C2.1	SbPP2C9	AtAHG1
3	3	1	OsPP2C49	SvPP2C6	SbPP2C4	*
2	2	1	OsPP2C50	SvPP2C9	*	*
4	4	1	OsPP2C53	SvPP2C1	SbPP2C2	AtABI2
3	3	1	OsPP2C68	SvPP2C4	SbPP2C3	*

# Species	Genes	Alg.-Conn.	Os_SnRK2	Sv_SnRK2	Sb_SnRK2	At_SnRK2
4	8	0.25	OsSAPK8,OsSAPK10	SvSnRK2.2,SvSnRK2.1, SvSnRK2.3	SbSnRK2.1,SbSnRK2.2	AtSnRK2.6,AtSnRK2.3
3	3	0.667	OsSAPK4	SvSnRK2.10	SbSnRK2.10	*
4	8	0.113	OsSAPK2,OsSAPK1	SvSnRK2.5,SvSnRK2.4.1	SbSnRK2.4,SbSnRK2.5	AtSnRK2.7,AtSnRK2.8
4	10	0.544	OsSAPK7,OsSAPK6	SvSnRK2.7, SvSnRK2.6	SbSnRK2.6,SbSnRK2.7	AtSnRK2.5,AtSnRK2.4,AtSnRK2.10,AtSnRK2.1
4	5	0.2	OsSAPK5	SvSnRK2.11, SvSnRK2.8	SbSnRK2.8	AtSnRK2.9
3	3	1	OsSAPK3	SvSnRK2.9	SbSnRK2.9	*
2	2	1	OsSAPK9	*	SbSnRK2.3	*

Table S2. Statistical analysis of physiological measurements during drought stress.

Drought stress									
		0h	24h	36h	39h	42h	45h	RW 2h	RW 4h
A10.1	*A*		***	***	***	***	***	***	***
	gs		***	***	***	***	***	***	*
	E		***	***	***	***	***	***	**
		0h	24h	36h	39h	42h	45h	RW 2h	RW 4h
Ast-1	*A*				***	***	***	***	***
	gs				***	***	***	***	***
	E				***	***	***	***	***

The asterisks represent (*$p > 0.05$; ** $p > 0.01$; ***$p > 0.001$) (non-statistically significant), unpaired t test.

Table S3. Statistical analysis of physiological measurements during salt stress.

Salt stress						
		0h	24h	48h	72h	96h
A10.1 100mM	*A*			*	**	**
	gs		**		***	**
	E		**		***	**
		0h	24h	48h	72h	96h
A10.1 200mM	*A*			*	***	***
	gs		*		***	***
	E				***	***
		0h	24h	48h	72h	96h
Ast-1 100mM	*A*				*	**
	gs				*	**
	E				*	**
		0h	24h	48h	72h	96h
Ast-1 200mM	*A*	*	**	***	***	***
	gs	*	**	***	***	***
	E	*	**	***	***	***

The asterisks represent (*$p > 0.05$; ** $p > 0.01$; ***$p > 0.001$) (non-statistically significant), unpaired t test.

Table S4. Statistical analysis of physiological measurements during cold stress.

Cold stress						
		25°C	20°C	15°C	10°C	5°C
A10.1	*A*			***	***	***
	gs			***	**	***
	E			***	***	***
		25°C	20°C	15°C	10°C	5°C
Ast-1	*A*	***	**	***	***	***
	gs	***	***	***	***	***
	E	***	***	***	***	***

The asterisks represent (*$p > 0.05$; ** $p > 0.01$; ***$p > 0.001$) (non-statistically significant), unpaired t test.

Table S5. Statistical analysis of physiological measurements during exogenous ABA application.

Exogenous ABA					
		0h	2h	4h	6h
A10.1 100µM	*A*			**	**
	gs			**	**
	E			**	**
		0h	2h	4h	6h
Ast-1 100µM	*A*				
	gs			**	**
	E		*	**	**
		0h	2h	4h	6h
Ast-1 200µM	*A*		**	**	***
	gs		***	***	***
	E		***	***	***

The asterisks represent (*$p > 0.05$; ** $p > 0.01$; ***$p > 0.001$) (non-statistically significant), unpaired t test.

Table S6. Sequence of the primer pairs used for RT-qPCR analysis.

Gene name	*S. viridis* Phytozome ID	Primer Sequences (5' - 3')
**PYL 1 ou PYL1**	Sv 9G318000	F: GCGAGGTCAGCGTCATC<br>R: GGTGATGGAGAAGCCGAAG
**PYL 2**	Sv 1G031000	F: GTTGGTGACATCGACTGAAATG<br>R: GACACTGCAGGGATAAGGATAG
**PYL 3**	Sv 4G251800	F: CGTCGTCGTCGAGTCCTA<br>R: TCTGGAGGTTGAGCCTGA
**PYL 4**	Sv 9G441100	F: TCGTCGTGGAGTCCTACAT<br>R: CTGGAGGTTGCACCTGAC
**PYL 5**	Sv3G213000	F: CGTGGTGGTCGAGTCGTA<br>R: CAGCGACTGGAGGTTGC
**PYL 6**	Sv 5G374800	F: AGTCCTACGTGGTGGATGTA<br>R: GACCCATAACCTTGCGTTCTA
**PYL 7**	Sv 3G077900	F: TGAGACATGCTACTTTGTCGAG<br>R: GAGCGGTGATGTAGGAGATTG
**PYL 8**	Sv 1G013800	F: GTGATCGAGTCCTTCGTTGTG<br>R: CGACTTGAGGTTGCACTTGA
**SnRK2.1**	Sv 9G078200	F: CTGGAGAACACACTGCTAGATG<br>R: GGTCACTCCACATGACCATAC
**SnRK2.2**	Sv 9G167300	F: CATTGCTCCTGAGGTTCTTCT<br>R: GATCTTGGCACTCTGGAGATATG
**SnRK2.3**	Sv 3G387400	F: TCCCGACTATGTCCATGTATCT<br>R: ACGAACCAAGGATGGCTTATT
**SnRK2.4.1**	Sv 9G384100.1	F: TGCACTGCTGGGAGATTTAG<br>R: GTGGTGCAACACTACCATCTA
**SnRK2.4.2**	Sv 9G384100.2	F: GAACCTGCCCATTGAGATGA<br>R: CTGGATGATCGCCATGACTT
**SnRK2.5**	Sv 2G405700	F: GTTGTGCTAACTCCCACACA<br>R: TTGGTCATCCTCGCTAAATCTC
**SnRK2.6**	Sv 1G194000	F: GGGAGAATTACGATTAGGGAGATTAG<br>R: GGGCACTGTTGTCCTTCTT
**SnRK2.7**	Sv .7G108300	F: CTGTGGAGTGACCCTTTATGT<br>R: GGTTCCTGATCTCCCTAATTGT
**SnRK2.8**	Sv 3G004100	F: GCAGTACAAGATTCCAGAGTATG<br>R: GGGTTTGCGACGAAGATG
**SnRK2.9**	Sv 9G323900	F: GAATATGCTGCTGGTGGAGAG<br>R: CAAGGAGGGTGTTCTCAAGTTTA
**SnRK2.10**	Sv 3G235900	F: GGGTGATCCTAACTCCAACAC<br>R: TTGCTCATCCTCGCTAAACC
**SnRK2.11**	Sv 5G400900	F: GTGGGAGCATACCCATTTGA<br>R: GGAACCATGGATGGCTCTTTA
**PP2C1**	Sv 3G124100	F: AGCCAAACAGGGAAGATGAG<br>R: GATCGGGACATGGCAAGAA
**PP2C2.1**	Sv 6G004900.1	F: GTGATCTACATCAACGGCCATC<br>R: ATCTCAGGCTCAGCGATCA
**PP2C2.2**	Sv 6G004900.2	F: ATGCGTGGCAGCAAGTTA<br>R: ATTCGGAAGGTGCTGGAATG
**PP2C3.1**	Sv 7G004500.1	F: GTCGAGCAAGGTGAAGAAGTAG<br>R: GCCGCCAGAACCTGATG
**PP2C3.2**	Sv 7G004500.2	F: GTCGAGCAAGGTGAAGAAGTAG<br>R: GCCGCCAGAACCTGATG
**PP2C4**	Sv 2G184100	F: GACGAGTGCCTCATCCTG<br>R: CTCAGCGCACCACTTCT
**PP2C5**	Sv 5G384400	F: GACGCAGGTTGGAGATGAG<br>R: TCGAACTCCGAGACCACTT

F: Forward; R: Reverse.

Table S6. Continued…

**PP2C6**	Sv 3G223900	F: GACGCCGTGTCCATCAG<br>R: CGTCAAAGACGCCAAAGAAAT
**PP2C7.1**	Sv 9G463500.1	F: CCATTGGCGACAGCTACC<br>R: AGGATCAGGAACTCGTCCTC
**PP2C7.2**	Sv 9G463500.2	F: CTGAAGCCGTTCGTGATCT<br>R: ACCTCGTTGCTCACCAC
**PP2C8**	Sv 1G014100.1	F: ACAGGATCGATGAGCGAAAG<br>R: CTAGCTAGAACCAAGCATTCCA
**PP2C9**	Sv 3G168300.1	F: GAGCCTGACAGAAAGGATGAG<br>R: TTGATCGCGACATAGCAAGTAT
**PP2C10**	Sv 5G211600	F: CTAACAGGGAGGATGAGTATGC<br>R: GACCGTGACATTGCAAGAAC
**PP2C12**	Sv3G141300	F: CCACTGATCACAAGGTGACTAC<br>R: TGGCCAGGATGAGGAACT
**CUL**	Sevir.3G038900	F: TCTCATCACGAGGGACTACTT<br>R: CTTGCCAACAACCACCAATC
**CAC**	Sevir.1G284400	F: CTGCTTCTGGTCTTCGTGTT<br>R: GTATGATCCTGCTCTCGTGATG
**EFL1**	Sevir.3G272400	F: TGGTATGCTTGTCACCTTTGGT<br>R: CTCGTGGTGCATCTCAACTGA
**EFL4**	Sevir.1G088000	F: GTCTGCTAAGGTGCTGGATAAA<br>R: ACCACTCCTCCAGAACATAGA
**SUI**	Sevir.2G348300	F: CCAGAGCTTGGACAGGTCATTTC<br>R: ACAATGCCAGCCTGGACAA

F: Forward; R: Reverse.
